# Phytochemicals and In Vitro Bioactivities of Aqueous Ethanolic Extracts from Common Vegetables in Thai Food

**DOI:** 10.3390/plants10081563

**Published:** 2021-07-29

**Authors:** Uthaiwan Suttisansanee, Parunya Thiyajai, Parisut Chalermchaiwat, Khanitha Wongwathanarat, Kanchana Pruesapan, Somsri Charoenkiatkul, Piya Temviriyanukul

**Affiliations:** 1 Institute of Nutrition, Mahidol University, Salaya, Phuttamonthon, Nakhon Pathom 73170, Thailand; uthaiwan.sut@mahidol.ac.th (U.S.); parunya.thy@mahidol.ac.th (P.T.); somsri.chr@mahidol.ac.th (S.C.); 2 Food and Nutrition Academic and Research Cluster, Institute of Nutrition, Mahidol University, Salaya, Phuttamonthon, Nakhon Pathom 73170, Thailand; 3 Food and Nutrition Program, Department of Home Economics, Faculty of Agriculture, Kasetsart University, Bangkok 10900, Thailand; fagrpsch@ku.ac.th; 4 Biotechnology Research and Development Office, Department of Agriculture, Ministry of Agriculture and Cooperatives, Bangkok 10900, Thailand; kwongwath@yahoo.com; 5 Plant Varieties Protection Division, Department of Agriculture, Ministry of Agriculture and Cooperatives, Bangkok 10900, Thailand; kpruesapan@gmail.com

**Keywords:** antioxidant activities, enzyme inhibitory activities, in vitro health properties, non-communicable diseases, phenolics, vegetables

## Abstract

Non-communicable diseases (NCDs) are the leading global cause of death. The World Health Organization (WHO) has endorsed the consumption of fruits and vegetables because they are rich in phytochemicals that sustainably ameliorate the occurrence of NCDs. Thai food contains many spices and vegetables with recognized health benefits. Quality control of plant samples encountered a bottleneck in the field and comparative studies of plant control origins including species or cultivar identification, growing area and appropriate harvesting time are limited. To address this issue, all plant samples used in this study were cultivated and controlled by the Department of Agriculture, Ministry of Agriculture and Cooperatives, Thailand. The samples were phytochemically screened and determined their health-promoting bioactivities via antioxidant activities and inhibition of NCD-related enzymes including lipase (obesity), α-amylase and α-glucosidase (diabetes), angiotensin-converting enzyme (hypertension), as well as acetylcholinesterase, butyrylcholinesterase and β-secretase (Alzheimer’s disease). The non-enzymatic reaction toward glycation was also evaluated. The results showed that *Senegalia pennata* subsp. *insuavis* (Lace) Maslin, Seigler & Ebinger, *Citrus hystrix* DC. and *Solanum melongena* ‘Kermit’ extracts exhibited high antioxidant activities. Moreover, *Citrus hystrix* DC. extract was a potent inhibitor against lipase, angiotensin-converting enzyme and butyrylcholinesterase, while *Coriandrum sativum* L. and *Psophocarpus tetragonolobus* (L.) DC. were potent anti-diabetic agents and *Senegalia pennata* subsp. *insuavis* (Lace) Maslin, and Seigler & Ebinger was a potent anti-glycation agent. Our data provide a comparative analysis of ten vegetables to encourage healthy food consumption and development to control NCDs in Thailand in the future.

## 1. Introduction

Non-communicable diseases (NCDs) including cancer, diabetes mellitus (type II), cardiovascular diseases, hypertension, and Alzheimer’s disease (AD) are the leading cause of death worldwide. In 2012, the World Health Organization (WHO) reported that NCDs accounted for 68% of morbidity (38 million deaths from 56 million deaths), while over 16 million (40%) were early deaths (<70 years) [1]. Risk factors involving NCDs include smoking, insufficient physical activity, alcohol, and unhealthy food consumption. Hence, the WHO has endorsed the benefits of fruit and vegetable consumption by recommending an intake of 400 g/person/day [2]. Previous studies demonstrated the health benefits of fruit and vegetable consumption. Borgi et al. (2016) [3] reported that long-term consumption of whole fruits and vegetables especially broccoli, carrots, soybeans, raisins, and apples reduced the risk of developing hypertension, while Chiavaroli et al. (2019) [4] examined systematic reviews and meta-analyses of dietary consumption of fruit, vegetables, whole grains, and low-fat dairy (Dietary Approaches to Stop Hypertension dietary pattern, DASH) and cardiometabolic disease outcomes. The results indicated that DASH dietary patterns reduced cardiovascular disease, diabetes, and stroke incidence. Metabolic syndromes and high intake of vitamins and phytonutrients including anthocyanidins, flavonols and flavonoids also reduced the risk of AD [5,6].

Fruits and vegetables are rich in fiber, vitamins, minerals, and phytochemicals including alkaloids, anthocyanins, glucosinolates, flavonoids, phytosterols, phenolic acids and terpenoids that are secondary plant metabolites, with pharmacological effects toward a wide range of ailments including NCDs [7]. Phytochemicals promote human health benefits through several mechanisms including being antioxidants [8], possessing anti-inflammatory activities [9], controlling or modulating signaling transduction against tumorigenesis in cells [10] and inhibiting key enzymes involved in the pathogenesis of diseases [11,12,13]. Intriguingly, modes of action of some current medicines against NCDs are based on their inhibitory functions against the responsible enzymes. Enzymes that are drug targets for NCDs include lipase (obesity), α-amylase and α-glucosidase (diabetes), angiotensin-converting enzyme (ACE) (hypertension) as well as acetylcholinesterase (AChE), butyrylcholinesterase (BChE) and β-secretase (BACE-1) (AD). Advanced glycation end products (AGEs), a group of molecules generated by a non-enzymatic glycation reaction between proteins and carbonyl compounds or reducing sugars, contribute to the prevalence of diabetes, neurodegenerative diseases, and aging [14]. Thus, inhibiting AGE formation would be beneficial for NCD prevention [15]. Plant extracts and phytochemicals have a proven potential to inhibit AGEs and glycation reactions; therefore, the consumption of fruits and vegetables offers a promising approach for preventing NCDs [11,12,13,16,17,18].

Thailand is renowned for its mouthwatering cuisine, consisting of a wide variety of textures and aromas emanating from local vegetable ingredients. Scientific reports have identified the health benefits of vegetables used in Thai dishes including antioxidant [19], anti-inflammatory [20], anti-obesity [21] and anti-hypertensive properties [22]. However, comparative studies are limited due to the lack of plant control origins such as species or cultivar identification, cultivation area and appropriate harvesting time. Moreover, even though some previous reports had indicated biological properties of these plants, our study is interested in different plant parts, which have been practically used in many Thai cuisines, some of which had never been investigated regarding their phytochemicals and bioactivities before. Thus, to address these issues, this study was undertaken to comparatively analyze the in vitro health-promoting activities of vegetables in Thai cuisine including *Allium cepa* Aggregatum Group, *Allium fistulosum* L., *Allium sativum* L., *Citrus hystrix* DC., *Coriandrum sativum* L., *Cymbopogon citratus* (DC.) Stapf, *Eryngium foetidum* L., *Psophocarpus tetragonolobus* (L.) DC., *Senegalia pennata* subsp. *insuavis* (Lace) Maslin, Seigler & Ebinger, and *Solanum melongena* ‘Kermit’ against NCDs. Despite having nothing in common (different species, plant parts, etc.), these plants have been selected based on their regular and frequent usages in most Thai cuisines. All plant samples were vetted for their origin and quality by the Department of Agriculture, Ministry of Agriculture and Cooperatives, Thailand. 

## 2. Results

### 2.1. Phytochemical Analyses

High-performance liquid chromatography (HPLC) was employed to determine the specific phytochemical profiles covering the flavonoids and phenolic acids of vegetable extracts including *Allium cepa* Aggregatum Group (*A. cepa*), *Allium fistulosum* L. (*A. fistulosum*), *Allium sativum* L. (*A. sativum*), *Citrus hystrix* DC. (*Ci. hystrix*), *Coriandrum sativum* L. (*Co. sativum*), *Cymbopogon citratus* (DC.) Stapf (*Cy. citratus*), *Eryngium foetidum* L. (*E. foetidum*), *Psophocarpus tetragonolobus* (L.) DC. (*P. tetragonolobus*), *Senegalia pennata* subsp. *insuavis* (Lace) Maslin, Seigler & Ebinger (*Se. pennata*), and *Solanum melongena* ‘Kermit’ (*So. melongena*). Seven flavonoids were detected: quercetin, kaempferol, hesperidin, luteolin, apigenin, delphinidin, and cyanidin (Table 1). Among the ten extracts, four (*A. cepa*, *Ci. hystrix*, *Co. sativum,* and *P. tetragonolobus*) contained two flavonoids at different concentrations. Four extracts possessed only one flavonoid: *A. fistulosum* (kaempferol), *Cy. citratus* (luteolin), *E. foetidum* (kaempferol), and *Se. pennata* (apigenin). Interestingly, no flavonoids were detected in *A. sativum* and *So. melongena* extracts. An aqueous ethanolic extract of *Co. sativum* was rich in quercetin (166.16 mg/100 g dry weight (DW)), followed by *A. cepa* and *Ci. hystrix* (63.34 and 25.52 mg/100 g DW, respectively), while kaempferol (4.44–47.97 mg/100 g DW) was detected in *A. fistulosum*, *Co. sativum*, and *E. foetidum* extracts, with *A. fistulosum* extract exhibiting the highest (47.97 mg/100 g DW). Hesperdin (453.47 mg/100 g DW) was solely detected in *Ci. hystrix* extract, luteolin (4.57 mg/100 g DW) in *Cy. citratus* extract, apigenin (3.46 mg/100 g DW) in *Se. pennata* extract, and delphinidin (15.77 mg/100 g DW) in *P. tetragonolobus* extract. Cyanidin was also found in *P. tetragonolobus* (43.02 mg/100 g DW) and *A. cepa* (13.35 mg/100 g DW).

For phenolic acid determination (Table 2), five phenolic acids including 4-hydroxybenzoic acid, vanillic acid, caffeic acid, *p*-coumaric acid, and ferulic acid were detected. Caffeic acid and *p*-coumaric acid were general phenolics as they were observed in six extracts with different concentrations. The highest content of caffeic acid (246.99 mg/100 g DW) was detected in *So. melongena* extract that also contained minute amounts of *p*-coumaric acid (2.16 mg/100 g DW). The highest content of *p*-coumaric acid (68.13 mg/100 g DW) was detected in *Cy. citratus* extract, which also possessed the highest content of ferulic acid (123.34 mg/100 g DW) and marginal amounts of caffeic acid (15.65 mg/100 g DW). The second most abundant caffeic acid (52.69 mg/100 g DW) was detected in *E. foetidum* extract, which also contained minute amounts of *p*-coumaric acid and ferulic acid (2.63 and 1.77 mg/100 g DW, respectively). An aqueous ethanolic extract of *P. tetragonolobus* contained the most varieties of phenolic acids including caffeic acid (16.13 mg/100 g DW), vanillic acid (15.71 mg/100 g DW), 4-hydroxybenzoic acid (10.80 mg/100 g DW), and *p*-coumaric acid (3.85 mg/100 g DW), while 4-hydroxybenzoic acid was only detected in *P. tetragonolobus* extract. Other than being observed in *P*. *tetragonolobus* extract, vanillic acid was also found in *Co. sativum* but in lower amounts (3.73 mg/100 g DW). This extract also contained caffeic acid (23.81 mg/100 g DW) and *p*-coumaric acid (5.20 mg/100 g DW). An aqueous ethanolic extract of *A. fistulosum* was found to possess ferulic acid (23.13 mg/100 g DW) and *p*-coumaric acid (7.12 mg/100 g DW), while *Se. pennata* contained only one phenolic acid: caffeic acid (14.92 mg/100 g DW). Interestingly, no phenolic acids were observed in *A. cepa*, *A. sativum*, and *Ci. hystrix* extracts.

A spectrophotometric analysis indicated that TPCs of all vegetable extracts ranged from 1.23 to 15.33 mg gallic acid equivalent (GAE)/g DW (Table 3). The aqueous ethanolic extract of *Se. pennata* exhibited the highest TPC, followed by *Ci. hystrix*, *So. melongena*, *Cy. citratus*, *A. fistulosum*, *P. tetragonolobus*, *E. foetidum*, *A. cepa*, and *Co. sativum*, respectively, while *A. sativum* exhibited the lowest (twelve times lower than *Se. pennata*). 

### 2.2. Antioxidant Activities

Phenolics contribute to a wide range of health benefits, including being antioxidants. Antioxidant activities were investigated covering both hydrogen atom transfer (HAT) and single electron transfer (SET) mechanisms (Table 3). The ferric reducing antioxidant power (FRAP) and 2,2-diphenyl-1-picrylhydrazyl (DPPH) radical scavenging assays were performed under the SET mechanism, while the oxygen radical absorbance capacity (ORAC) assay followed the HAT mechanism. The DPPH radical scavenging activities ranged from 1.77 to 33.06 μmol Trolox equivalent (TE)/g DW with *Se. pennata* extract exhibiting the highest DPPH radical scavenging activity and *A. sativum* extract the lowest. For the FRAP assay, results were consistent with the DPPH radical scavenging activities. *Se. pennata* exhibited the highest reducing ability (62.33 μmol TE/g DW) of ferric iron (Fe^3+^) to ferrous iron (Fe^2+^), while *A. sativum* exhibited the lowest reducing activity (3.25 μmol TE/g DW). On the other hand, *Ci. hystrix* and *So. melongena* possessed the highest ORAC activities (415.92–418.32 μmol TE/g DW), while *A. cepa* exhibited the lowest (15.12 μmol TE/g DW) at approximately 27-fold lower than *Ci. hystrix* and *So. melongena*. 

### 2.3. Enzyme- and Non-Enzyme Inhibitory Activities 

All the vegetable extracts contained phenolic acids and flavonoids that contributed to their therapeutic potential, specifically against some NCDs. Therefore, all the extracts were tested for their therapeutic potential against critical enzymes involved in NCDs and non-enzymatic reactions involving anti-glycation properties. 

Lipase is a lipid-degradation enzyme, and lipase inhibitors prevent fatty acid accumulation as one characteristic of obesity [23]. The results showed that all vegetable extracts inhibited lipase, while inhibitory activities ranged from 12.9 to 61.2% using an extract concentration of 1 mg/mL (Table 4). The aqueous ethanolic extract of *Ci. hystrix* exhibited the highest lipase inhibition, while *A. cepa* exhibited the lowest.

Type II diabetes is known for high blood glucose levels; hence, slowing down carbohydrate digestion could be one of the therapeutic targets for diabetes. Two enzymes including α-amylase and α-glucosidase function in carbohydrate digestion [24]. The former breaks down starch into disaccharides and trisaccharides, while the latter hydrolyzes disaccharides into glucose. Although the extracts displayed anti-α-amylase activities ranging from 4.1 to 58.4% using an extract concentration of 1 mg/mL, only three extracts including *Co. sativum* (58.4%), *E. foetidum* (31.2%), and *Ci. hystrix* (26.6%) showed inhibitory activities of more than 25% (Table 4). For α-glucosidase inhibition, all extracts except *A. sativum* inhibited α-glucosidase reaction in the range of 8.1–64.0% using an extract concentration of 1 mg/mL. Among extracts with α-glucosidase inhibitory activities, the highest inhibition was detected in *P. tetragonolobus* with the lowest in *A. cepa*. Thus, *Co. sativum* and *P. tetragonolobus* may be potential candidates with high anti-diabetic properties via the reduction of sugar generated from carbohydrate degradation.

Angiotensin-converting enzyme (ACE) converts angiotensin I to angiotensin II, leading to vasoconstriction and increased blood pressure [16]; thereby, ACE inhibition could reduce the risk of hypertension. Interestingly, all vegetable extracts acted as effective ACE inhibitors, with inhibitions ranging from 50.4 to 91.7% using extract concentration of 0.2 mg/mL (Table 4). The three extracts with the highest ACE inhibitions were *A. cepa*, *Ci. hystrix*, and *Se. pennata*, while the lowest inhibition was observed in *So. melongena*.

Causes of Alzheimer’s disease (AD), one type of dementia, have been attributed to (i) degradation of the neurotransmitter acetylcholine by acetylcholinesterase (AChE) and butyrylcholinesterase (BChE), or (ii) accumulation of amyloid plaque formed during amyloidogenesis by β-secretase (BACE-1) [25]. Therefore, these enzymes are targeted for anti-AD drug development. All vegetable extracts, except *A. sativum*, inhibited AChE activities in the range of 8.3–58.6% using extract concentration of 1 mg/mL (Table 4). Among the extracts with inhibitory activities, *E. foetidum* displayed the highest inhibition, while *A. cepa* was the lowest. All extracts inhibited BChE activities in the range of 3.7–52.6% using the same extract concentration (Table 4). The highest inhibition was observed in *Ci. hystrix*, while the lowest was in *So. melongena*. For BACE-1 inhibitory activities, all extracts, except *A. sativum*, inhibited BACE-1 in the range of 12.5–39.1% using extract concentration of 1 mg/mL (Table 4). Among the extracts with inhibitory activities, the highest inhibition was observed in *A. fistulosum*, while the lowest was *Cy. citratus*. Overall, *Ci. hystrix* and *E. foetidum* showed potential as candidates for AD treatment via the reduction of acetylcholine, while *A. fistulosum* showed promise via amyloid plaque formation. However, *A. sativum* showed deficient inhibitory activities toward AD-related enzymes in this study, with no inhibitory activities detected in AChE and BACE-1 reactions and low inhibitory activities detected in BChE reaction.

The glycation reaction is a non-enzymatic reaction involving interaction between monosaccharides and amino acids, lipids, or nucleotides. Methylglyoxal (MG), as a by-product of glycolysis, can act as a potent protein-glycation inducing agent. Finally, glycation induced by either D-glucose or MG as advanced glycation end products (AGEs) contributed to several ailments including premature aging and diabetes [26]. The results indicated that all vegetable extracts prevented glycation reaction induced by either D-glucose (10.6–74.6%) or MG (5.1–81.5%) using an extract concentration of 0.63 mg/mL (Table 5). Interestingly, *Se. pennata* provided the most potent anti-glycation activities induced by both D-glucose and MG, while *A. sativum* and *A. cepa* exhibited poor ability to inhibit glycation reactions.

### 2.4. Correlation by Principal Component Analysis (PCA) and Hierarchical Cluster Analysis (HCA)

**Figure 1:**
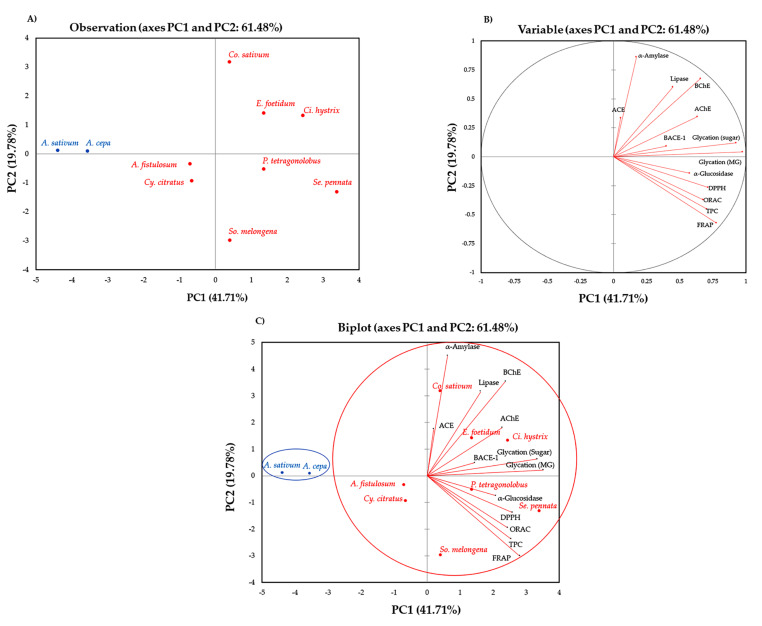
Principal component analysis (PCA) from mean values of all variables of ten vegetable extracts: (**A**) observation, (**B**) variable, and (**C**) biplot.

Relationships between vegetable extracts and TPCs, antioxidant activities, enzyme inhibitory activities and anti-glycation properties were investigated using principal component analysis (PCA) and hierarchical cluster analysis (HCA) to determine the particular characteristics of each vegetable extract. Information gained from these statistical analyses will be helpful to classify Thai vegetables according to their unique health-promoting characteristics. PCA results showed that TPCs, antioxidant activities, enzyme inhibitory activities, and anti-glycation properties of Thai vegetable extracts could be easily classified. (Figure 1). 

Figure 1A shows a relationship among observations (taxa of ten Thai vegetables), and it was noticed that *A. cepa* and *A. sativum* (blue letters) were separated from others (red letters). Figure 1B shows a relationship among thirteen variables (TPCs, antioxidant activities, enzyme inhibitory activities, and anti-glycation properties). The first two axes (PCs) explained 61.48% of the total variance. PC1 (41.71%) was closely related to TPCs, antioxidant activities (determined by DPPH radical scavenging, FRAP, and ORAC assays), enzyme inhibitory activities (α-glucosidase and AChE inhibitory activities), and anti-glycation properties (induced by both D-glucose and MG), while PC2 (19.78%) was associated with enzyme inhibitory activities including lipase, α-amylase, and BChE. However, ACE was located in PC3 (the first two axes (PC1 and PC3) explained 53.77% of the total variance, while PC1 was 41.71% and PC3 was 12.07%). The data suggested that ACE inhibitory activity was unrelated from others. The biplot of PCA (Figure 1C) shows that the samples with different projected directions had different characteristics. It clearly observed that *A. cepa* and *A. sativum* (Cluster 1) were separated from the others (cluster 2) and located far away from the centroid (Figure 1A,C), indicating that these two vegetables had a minor relationship with the others, which could be due to their bioactive constituents and their poor enzyme inhibitory activities. 

**Figure 2:**
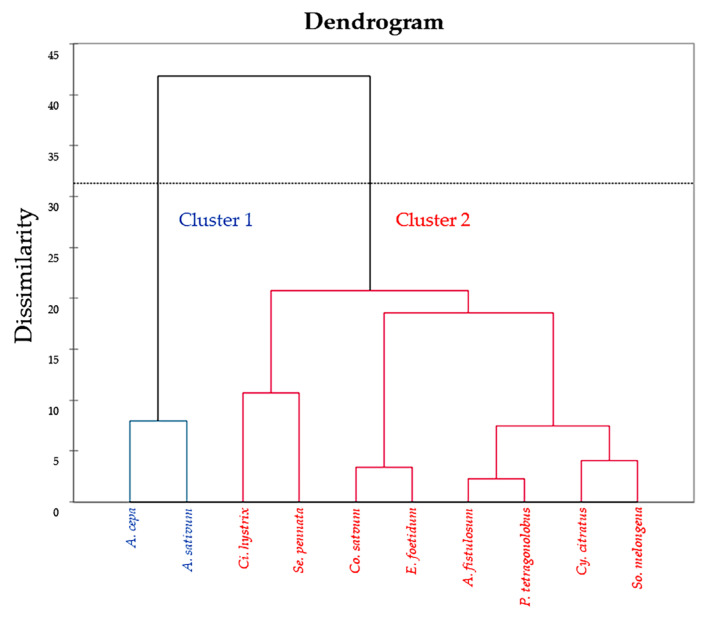
The dendrogram of hierarchical cluster analysis (HCA) of ten vegetable extracts.

HCA operates as an algorithm that assembles similar objects into clusters, as shown in Figure 2. The horizontal axis represents the clusters. The vertical scale on the dendrogram represent the distance or dissimilarity. Hence, if the distance (dissimilarity) between the two objects is small, they are considered to be in the same cluster. Objects within each cluster are estimated to be similar to each other in the group. Thus, hierarchical clustering is easy to implement, while the dendrogram produced is useful to understand and interpret the results [27]. In the present study, HCA results were classified between vegetable extracts and TPCs, antioxidant activities, enzyme inhibitory activities, and anti-glycation properties. The dendrogram shows two clusters. Cluster 1 consisted of *A. cepa* and *A. sativum* (blue color), while cluster 2 consisted of *A. fistulosum*, *Ci. hystrix*, *Co. sativum*, *Cy. citratus*, *E. foetidum*, *P. tetragonolobus*, *Se. pennata*, and *So. melongena* (red color). The outcome of HCA supported the PCA results (Figure 1C).

## 3. Discussion

Interest in indigenous plants for their health benefits in terms of disease prevention beyond nutritional benefits is increasing. Thai cuisine consists of various spices and herbs with unique aromas and flavors that also have health-promoting bioactivities. Copious literature exists on plant beneficial health characteristics, but control of plant origins is lacking, leading to the absence of a comparative analysis of these vegetables. Here, ten vegetables used in Thai cuisine including *Allium cepa* Aggregatum Group (*A. cepa*), *Allium fistulosum* L. (*A. fistulosum*), *Allium sativum* L. (*A. sativum*), *Citrus hystrix* DC. (*Ci. hystrix*), *Coriandrum sativum* L. (*Co. sativum*), *Cymbopogon citratus* (DC.) Stapf (*Cy. citratus*), *Eryngium foetidum* L. (*E. foetidum*), *Psophocarpus tetragonolobus* (L.) DC. (*P. tetragonolobus*), *Senegalia pennata* subsp. *insuavis* (Lace) Maslin, Seigler & Ebinger (*Se. pennata*), and *Solanum melongena* ‘Kermit’ (*So. melongena*) were comparatively analyzed regarding their phenolic profiles (phenolic acids and flavonoids) and in vitro inhibitory activities against some NCDs. The health-promoting activities involved the inhibition of the key enzymes that control NCDs including lipase (obesity), α-amylase, and α-glucosidase (diabetes), angiotensin-converting enzyme (hypertension) and acetylcholinesterase, butyrylcholinesterase, and β-secretase (Alzheimer’s disease) as well as the non-enzymatic anti-glycation reaction (premature aging). Results showed that among these ten plant extracts, *A. cepa* exhibited the strongest angiotensin-converting enzyme (ACE) inhibition, while *A. fistulosum* could effectively fight against β-secretase (BACE-1). Nevertheless, *A. sativum* seemed to be the least active extract against these NCDs-related enzymes, in which no inhibitory activities against α–glucosidase, acetylcholinesterase (AChE), and BACE-1 were observed. However, high ACE inhibitory activity was observed in this plant extract, while other enzyme inhibitory activities were quite low (less than 50% inhibition). On the other hand, *Ci. hystrix* was the most active extract, which exhibited the strongest antioxidant activity and inhibitory activities against lipase, ACE and butyrylcholinesterase (BChE). *Co. sativum* was highly effective against α-amylase, while *P. tetragonolobus* exhibited the highest α-glucosidase inhibitory activity. *Cy. citratus* seemed to exhibit low to moderate enzyme inhibitory activities, with the exception of ACE inhibitory activity, which was more than 50% inhibition. *E. foetidum* exhibited the highest AChE inhibitory activity, while *Se. pennata* exhibited strong antioxidant activity and ACE inhibitory activity. Similarly, *So. melongena* was also a good source of antioxidants. From these results, it is of interest to group these plants according to their bioactivities and discuss the following topics in more detail; (i) *Se. pennata* exhibited the highest reducing and free radical scavenging abilities, while *Ci. hystrix* and *So. melongena* possessed the highest oxygen radical absorbance capacity; (ii) *Ci. hystrix* exhibited the highest lipase inhibition; (iii) *Co. sativum* and *P. tetragonolobus* were potential anti-diabetic agents with high inhibitions against carbohydrate-degrading enzymes; (iv) *A. cepa*, *Ci. hystrix*, and *Se. pennata* had the three highest ACE inhibitory activities; (v) *Ci. hystrix* and *E. foetidum* were potential anti-Alzheimer’s disease (AD) agents with high inhibitions against acetylcholine degrading enzymes, whereas *A. fistulosum* acted against amyloid generating enzyme, and (vi) *Se. pennata* was a potential anti-glycation agent.

Among the vegetable extracts, *Se. pennata*, *Ci. Hystrix*, and *So. melongena* exhibited high antioxidant capacities with the highest total phenolic contents (TPCs). Our results concurred with previous literature suggesting that TPCs and antioxidant activities in various plant extracts were strongly correlated [12,28]. Young leaves and shoots of *Se. pennata* (or Cha-om in Thai) exhibit strong odor (some define as stinky) and are normally consumed as a blanched vegetable or mixed in an omelet and eaten with spicy sauce. In our study, *Se. pennata* exhibited high ferric reducing antioxidant power (FRAP) and 2,2-diphenyl-1-picrylhydrazyl (DPPH) radical scavenging activities, suggesting that antioxidants in this vegetable extract likely possess an ability to transfer one electron to any potential electron acceptors. A previous report suggested that methanolic extract of *Se. pennata* leaves exhibited high TPCs of 45.3 µg gallic acid equivalent (GAE)/mg dry extract with half maximal effective concentration (EC_50_) of 3.6 mg extract/mg DPPH [29], while its ethanolic extract of twig exhibited DPPH radical scavenging activity with half maximal inhibitory concentration (IC_50_) of 2.0 mg/mL [30]. Our study also found that *Ci. hystrix* and *So. melongena* exhibited high oxygen radical absorbance capacity (ORAC), suggesting that the antioxidants in these vegetable extracts likely donate hydrogen atoms to free radicals. The fruit peel of *Ci. hystrix* (also known as kaffir lime) is commonly used in many Thai recipes (such as chili paste and curry) for its strong and unique aroma. Eggplants in Thailand can be classified into 22 species of *Solanum*, with 10 cultivars commercially available [31]. Fruits of *So. melongena* can be consumed as fresh or blanched vegetables and are the main ingredient in curry. Previous literature also reported high TPCs and antioxidant activities of these two vegetables. Ethanolic extracts of *Ci. hystrix* fruit peel were previously reported to exhibit TPCs of 0.32 mg GAE/mg extract and DPPH radical scavenging activity with IC_50_ of 0.09 mg/mL [32]. Likewise, ethanolic extracts of fruits from different cultivars of *So. melongena* (cv. ‘Makhuea pro chao phraya’, cv. ‘Makhuea pro look lai’, and cv. ‘Makhuea pro muang’) exhibited DPPH radical scavenging activities of 17.52–46.13% [31].

Many authors have reported on the biological activities of *Ci. hystrix* but not on its anti-obesity property. Interestingly, in this study, fruit peel extract of *Ci. hystrix* was found to exhibit the highest lipase inhibition, an activity that retards lipid absorption and, thus, is related to the control of obesity. As the most abundantly found flavonoid in fruit peel of *Ci. hystrix*, hesperidin exhibited IC_50_ activity of 52.4 µM against porcine pancreatic lipase [33], while another flavonoid, quercetin, exhibited IC_50_ of 6.1 µM [34]. Compared to orlistat, a commercially available anti-lipase agent with an IC_50_ of 4 µM [34], quercetin is considered a strong lipase inhibitor. Even though phenolic acids were not detected in *Ci. hystrix*, it was previously found that phenolic acids were generally less active against lipase inhibition than flavonoids [34,35]. Therefore, among all the ten vegetable extracts, *Ci. hystrix* with the highest content of hesperidin and moderate amounts of quercetin possessed a potential bioactivity against lipase. 

As vegetable extracts with potential anti-diabetic properties, *Co. sativum* and *P. tetragonolobus* effectively inhibited α-amylase and α-glucosidase, respectively. Leaves and young shoots of *Co. sativum* (coriander) are added to many Thai dishes including soup or stir fry meat as a decorated vegetable with a strong unique aroma. However, recently, coriander has become popular consumed as a fresh side dish vegetable along with other main dishes. Oral administration of the ethanolic leaf extract to mice under induced insulin deficiency resulted in lowered blood glucose [34,35,36]. This ethanolic extract also effectively inhibited α-glucosidase with IC_50_ value 2.5-times lower than acarbose, a synthetic anti-diabetic drug [36]. Ethanolic extract of *Co. sativum* leaves inhibited α-amylase with 19% inhibition using extract concentration of 1 mg/mL [37]. Compared to acarbose with IC_50_ of 14.60 µM against pancreatic α-amylase, the most abundant flavonoid in *Co. sativum* leaves as quercetin exhibited IC_50_ of 12.7 µM [34], while the major phenolic acid, caffeic acid, exhibited IC_50_ of 20.4 µM [38]. With high quercetin and caffeic acid contents that act as strong α-amylase inhibitors, *Co. sativum* is a good candidate as an anti-diabetic agent, even its seeds [39,40,41]. Likewise, *P. tetragonolobus* (or winged bean) is a Thai local vegetable normally consumed as boiled or blanched young bean pod with many spicy sauces. At present, no report on young bean pod of *P. tetragonolobus* regarding its anti-diabetic property is available. Nevertheless, major phenolics in *P. tetragonolobus* such as cyanidin and delphinidin effectively inhibited *Saccharomyces cerevisiae* α-glucosidase with IC_50_ values of 17.0 and 4.1 µM, respectively, compared to acarbose with IC_50_ of 0.53 µM [42]. *P. tetragonolobus* also contained moderate contents of phenolic acids including 4-hydroxybenzoic acid, vanillic acid, caffeic acid and *p*-coumaric acid, while phenolic acids generally inhibited α-glucosidase with a lesser effect than flavonoids [42,43,44]. Comparison of enzyme inhibitions of flavonoids against α-glucosidase and α-amylase suggested that flavonoids affected α-glucosidase more effectively than α-amylase. Flavonoids fit more snuggly into active sites of α-glucosidase than α-amylase, thus making the former a better target enzyme for the prevention and treatment of diabetes [42]. 

Interestingly, all vegetable extracts effectively inhibited ACE at more than 50% inhibition using extract concentration of 1 mg/mL. Among these, *A. cepa*, *Ci. hystrix* and *Se. pennata* had the three highest ACE inhibitory activities. These results concurred with previous reports indicating that quercetin-rich onion skin extract decreased ambulatory blood pressure in patients under metabolic syndrome (overweight/obese/hypertension) [45]. The predominant flavonoid in *A. cepa* as quercetin exhibited IC_50_ of 43 µM against rabbit lung ACE [46], while hesperidin, the major flavonoid detected in *Ci. hystrix*, exhibited half inhibitory activity of quercetin using a concentration of 0.5 mM [46]. Caffeic acid was the major phenolic found in *Se. pennata* (but in low amounts compared to other vegetables) with IC_50_ of 5.7 mM [47].

For anti-AD properties, our data showed that using an extract concentration of 1 mg/mL, *Ci. hystrix* and *E. foetidum* acted as inhibitors for acetylcholine-degrading enzymes, while *A. fistulosum* showed promise as a potential extract inhibiting amyloid production. *Ci. hystrix* and *E. foetidum* were rich in hesperidin and caffeic acid, respectively, while the consumption of hespiridin-rich extract or caffeic acid avoided cognitive dysfunction and learning deficit in vivo [48,49]. Hesperidin exhibited IC_50_ against AChE and BChE at 22.8 and 48.9 µM, respectively, and caffeic acid at 23.36 and 29.19 µM, respectively [50,51]. Thus, hesperidin and caffeic acid play roles as inhibitors for both acetylcholine degrading enzymes, AChE and BChE. Interestingly, no report on the anti-AD properties of *E. foetidum*, usually used in the famous sour soup called “Tom Yum” was available; thus, future studies on the anti-AD properties of the caffeic acid rich extract of *E. foetidum* are required. When considering anti-AD via the inhibition of amyloid production, all extracts displayed mild to low BACE-1 inhibition. *A. fistulosum* and *A. cepa* gave the two highest anti-BACE-1 activities, albeit carrying different phytochemicals. *A. cepa* showed high quercetin, while *A. fistulosum* was high in kaempferol. Quercetin and kaempferol are well-known flavonoids exhibiting anti-BACE-1 properties with IC_50_ values at 5.4 and 14.7 µM, respectively [52]. Leaves of *Co. sativum* also possessed high quercetin (2.5-fold higher than *A. cepa*); however, the anti-BACE-1 activity was lower, indicating that multi-interaction of phytochemicals within the extract may display antagonist effects. 

The glycation reaction leads to the formation of advanced glycation end products (AGEs) that contribute to diseases such as diabetes, AD, and premature aging [26]. The reaction can be induced by either sugar or methylglyoxal (MG). Hence, besides key enzyme inhibition, inhibition of the glycation reaction might also be an effective strategy cooperating with enzyme inhibitors to reduce or prevent these diseases. Our data showed that *Se. pennata* may be a potential anti-glycation agent for both reactions. *Se. pennata* is typically fried with egg or with “Tom Yum” soup. The glycation reaction and its AGEs relate to free radical productions [53]. Hence, antioxidant compounds may be associated with anti-glycation properties. Among the extracts, *Se. pennata* exhibited high antioxidant activity covering the SET mechanism due to its high phenolics content. It remains unclear which phytochemicals in *Se. pennata* exhibit this property because only trace amounts of caffeic acid and apigenin were observed, even though these two compounds were documented for their anti-glycation properties [54,55].

Additionally, the principal component analysis (PCA) suggested that the activities that lied in the same axis were closely related to each other. For example, TPCs, antioxidant activities, α-glucosidase inhibitory activities, AChE inhibitory activities, and anti-glycation properties were on the same axis (PC1). The extract with relatively high TPCs and antioxidant activities would potentially exhibit relatively high anti-α-glucosidase, AChE, or glycation activities as well (i.e., *Ci. hystrix*). However, ACE inhibitory activity was different from other bioactivities since it was located in a different axis (PC3). Moreover, ACE inhibitory activities of all vegetable extracts were higher than 50%, even though the extract concentration used in this enzyme inhibitory assay was lower (0.2 mg/mL) than others (1 mg/mL in other enzyme inhibitory assays and 0.63 mg/mL in glycation reactions). It was previously reported that other than phenolics that could act as ACE inhibitors, small peptides could also act as effective ACE inhibitors as well [56]; it was possible that high ACE inhibitory activities observed in these vegetable extracts might be a biological function of both phenolics and small peptides. Furthermore, the biplot of PCA divided the vegetable extracts into two clusters with different bioactivities, the results of which corresponded with the hierarchical cluster analysis (HCA). Cluster 1 consisted of *A. cepa* and *A. sativum* exhibited low TPCs, antioxidant activities, and anti-glycation properties. Cluster 2 consisted of *A. fistulosum*, *Ci. hystrix*, *Co. sativum*, *Cy. citratus*, *E. foetidum*, *P. tetragonolobus*, *Se. pennata*, and *So. melongena* exhibited high TPCs, antioxidant activities, and enzyme inhibitory activities. 

In conclusion, this is the first comparative investigation on the phytochemicals and in vitro health-promoting activities of ten vegetables in Thai cuisine to potentially combat NCDs. Each plant possessed specific bioactivities, which can be grouped according to their capacities as follows. *Se. pennata*, *Ci. hystrix*, and *So. melongena* are good sources of antioxidants, while *Ci. hystrix* is also a good source of lipase inhibitors. *Co. sativum* and *P. tetragonolobus* provided effective inhibitors of carbohydrate degrading enzymes. *A. cepa*, *Ci. hystrix*, and *Se. pennata* are the top three with the highest ACE inhibition. *Ci. hystrix*, *E. foetidum,* and *A. fistulosum* could effectively fight against the key enzymes involved in AD, while *Se. pennata* provided powerful anti-glycation agents. However, since our study was performed using in vitro assays, further research is required to delineate the efficacy of the extracts in vivo more accurately. 

## 4. Materials and Methods

### 4.1. Sample Collection, Preparation, and Extraction

Ten vegetables including *Allium cepa* Aggregatum Group (*A. cepa*), *Allium fistulosum* L. (*A. fistulosum*), *Allium sativum* L. (*A. sativum*), *Citrus hystrix* DC. (*Ci. hystrix*), *Coriandrum sativum* L. (*Co. sativum*), *Cymbopogon citratus* (DC.) Stapf (*Cy. citratus*), *Eryngium foetidum* L. (*E. foetidum*), *Psophocarpus tetragonolobus* (L.) DC. (*P. tetragonolobus*), *Senegalia pennata* subsp. *insuavis* (Lace) Maslin, Seigler & Ebinger (*Se. pennata*), and *Solanum melongena* ‘Kermit’ (*So. melongena*) were collected following the recommendation of the Department of Agriculture, Ministry of Agriculture and Cooperatives, Thailand. The samples were deposited at the Bangkok Herbarium (BK), Bangkok, Thailand. Physical appearance of the edible part, harvesting time and the herbarium voucher specimen are provided in Table S1. Fresh edible parts of *A. cepa* (bulps), *A. fistulosum* (leaves), *A. sativum* (bulps), *Ci. hystrix* (fruit peel), *Co. sativum* (leaves), *Cy. citratus* (stalk), *E. foetidum* (leaves), *P. tetragonolobus* (whole fruits), *Se. pennata* (young leaves), and *So. melongena* (whole fruits) were cleaned with deionized (DI) water before freeze-drying using a Heto PowerDry PL9000 Freeze Dryer (Heto Lab Equipment, Allerod, Denmark) for 3 days. The dry samples were then ground into fine powder using a Philips 600W Grinder (Philips Electronics Co., Ltd., Jakarta, Indonesia. Colors of the fresh samples were determined using a ColorFlex EZ Spectrophotometer (Hunter Associates Laboratory, Reston, VA, USA) and expressed as CIELAB units (L* represented dark (0) to white (100), a* represented green (−) to red (+), while b* represented blue (−) to yellow (+)) as shown in Table S2. This color analysis was used as one indicator of sample quality control since maturity stage could be indicated by color change.

The dry samples were extracted using 80% (*v/v*) aqueous ethanol (1:10 ratio) at 37 °C for two hours. The mixture was centrifuged at 3800 *g* for 15 min using a Hettich^®^ ROTINA 38R centrifuge (Andreas Hettich GmbH, Tuttlingen, Germany). The supernatant was collected, while the residue was repeatedly extracted with the same procedure twice. The supernatants from three extractions were pooled, and ethanol was removed by a rotary evaporator (Eyela N-1200 Series, Eyela, Shanghai, China). The dried extracts were re-dissolved in DMSO, filtered through a 0.45 µM polytetrafluoroethylene (PTFE) membrane syringe filter, and kept at −20 °C until analysis. 

### 4.2. Determination of Phenolics Profile and Total Phenolic Contents

To determine the phenolic profile, high-performance liquid chromatography (HPLC) was employed using an Agilent 1100 HPLC system equipped with a photodiode array detector and a Zorbax Eclipse XDB–C18 column (150 × 4.6 mm, Agilent Technologies, Santa Clara, CA, USA) as previously described [13]. In brief, dry sample (0.5 g) was dissolved in the solvent containing 62.5% (*v/v*) aqueous methanol (40 mL), 6 N HCl (10 mL) and 0.5 g/L tert-butylhydroquinone (tBHQ). Prior to injection into the HPLC system, the extract (10 mg/mL) was filtered through a 0.22 μM PTFE membrane. Milli-Q water (18.2 MΩ.cm resistivity at 25 °C), HPLC-grade methanol, and HPLC-grade acetonitrile containing 0.05% (*v/v*) trifluoroacetic (TFA) were used as gradient mobile phases with a constant flow rate of 0.6 mL/min [13]. The authentic phenolic acid standards including 4-hydroxybenzoic acid (>99.0% GC, T), caffeic acid (>98.0% HPLC, T), chlorogenic acid (>98.0% HPLC, T), ferulic acid (>98.0% GC, T), *p*-coumaric acid (>98.0% GC, T), sinapic acid (>99.0% GC, T), and syringic acid (>97.0% T) were received from Tokyo Chemical Industry (Tokyo, Japan), while vanillic acid (≥97% HPLC) and gallic acid (97.5–102.5% T) were received from Sigma-Aldrich (St. Louis, MO, USA). The authentic flavonoid standards including quercetin (>98.0% HPLC, E), kaempferol (>97.0% HPLC), luteolin (>98.0% HPLC), hesperidin (>90.0% HPLC, T), naringenin (>93.0% HPLC, T), myricetin (>97.0% HPLC), and apigenin (>98.0% HPLC) were obtained from Tokyo Chemical Industry (Tokyo, Japan), while isorhamnetin (≥99.0% HPLC), cyanidin (≥96.0% HPLC), and delphinidin (≥97.0% HPLC) were from Extrasynthese (Genay, France). The phenolic acids were detected at 280 nm and 325 nm, while flavonoids were detected at 338 nm and 368 nm. HPLC chromatograms were shown in Figures S1–S3.

Linear range, linear regression, correlation coefficients, limit of quantitation (LOQ), limit of detection (LOD), and relative standard deviation (RSD) of the standards were analyzed according to the protocol of Srinuanchai et al. 2019 [57] as shown in Table S3. The LOQ and LOD were analyzed from the linear calibration curve with the equation as follows:

*y* = *a* + *bx*,

where *y* is an area under the peak, *a* is a *y*-intercept, *b* is a slope of the calibration curve, and *x* is a standard concentration. LOD and LOQ were calculated using the following equation:

LOQ = 10*S_a_*/*b* and LOD = 3.3*S_a_/b*,

where *S_a_* is a standard deviation of the response (*y*-intercept), and *b* is a slope of the calibration curve. The intra-day precision was presented as a percentage of the relative standard deviation (%RSD) and calculated using the following equation:

%RSD = 100 × (*S_tR_*/*Mean_tR_*),

where *S_tR_* is a standard deviation of the retention time, and *Mean_tR_* is the mean of the retention time measured at all concentrations of each standard.

Total phenolic contents (TPCs) were investigated using Folin’s phenol reagent as formerly described [58]. Gallic acid (up to 200 μg/mL) was used as a standard. The extracts (50 mg/mL) were diluted until they fitted within the linear range of the standard curve, and the TPCs were reported as mg gallic acid equivalent (GAE)/g dry weight. 

### 4.3. Determination of Antioxidant Activities

Antioxidant activities of the extracts were determined using 2,2-diphenyl-1-picrylhydrazyl (DPPH) together with ferric reducing antioxidant power (FRAP) and oxygen radical absorbance capacity (ORAC) assays as previously described [59]. Trolox, a water-soluble analog of vitamin E, was used as a standard. The extracts (50 mg/mL) were diluted until they fitted within the linear range of the standard curve, and antioxidant activities were reported as μmol Trolox equivalent (TE)/g dry weight.

### 4.4. Determination of Enzyme and Non-Enzyme Inhibitory Activities Using Spectrophotometric Techniques

To determine the enzyme inhibitory activities of the extracts against some NCDs, key enzymes that control obesity (lipase), diabetes (α-amylase and α-glucosidase), hypertension (angiotensin-converting enzyme), and Alzheimer’s disease (acetylcholinesterase, butyrylcholinesterase and β-secretase) were chosen for inhibitory reactions using the well-established protocols as previously described [11,12,13,22]. The inhibition of glycation reaction induced by D-glucose and methylglyoxal (MG) as a non-enzymatic reaction was also determined for anti-aging property [60]. Enzyme inhibitory assays consisted of an enzyme, a substrate, an indicator from Sigma-Aldrich (St. Louis, MO, USA) and a sample extract as an inhibitor. The enzyme inhibitory reaction was visualized using a Synergy^TM^ HT 96-well UV-visible microplate reader and Gen5 data analysis software (BioTek Instruments, Inc., Winooski, VT, USA). 

Briefly, the lipase inhibitory reaction consisted of 100 µL of 0.01 mg/mL *Candida rugosa* lipase (tуре VII, ≥700 unit/mg), 50 µL of 0.2 mM 5-5′-dithiobis(2-nitrobenzoic-*N*-phenacyl-4,5-dimethyуhiаzolium bromide), 10 µL of 16 mM 5,5′-dithiobis(2-nitrobenzoic acid) (DTNB) and 40 µL of the extract (5 mg/mL). The inhibitory activity was visualized as a decline in enzyme kinetics at 412 nm.

The α-amylase inhibitory reaction consisted of 100 µL of 30 mg/mL porcine pancreatic α-amylase (tуре VII, ≥10 unit/mg), 50 µL of 30 mM *p*-nitrophenyl-α-D-maltopentaoside and 50 µL of the extract (4 mg/mL), while the α-glucosidase inhibitory reaction consisted of 100 µL of 0.1 U/mL *Saccharomyces cerevisiae* α-glucosidase (type I, ≥10 U/mg protein), 50 µL of 2 mM *p*-nitrophenyl-α-D-glucopyranoside and 50 µL of the extract (4 mg/mL). The inhibitory activity was visualized as a decline in enzyme kinetics at 405 nm.

The angiotensin-converting enzyme (ACE) inhibitory reaction consisted of 3 µL of 0.5 U/mL rabbit lung ACE (≥2 unit/mg), 30 µL of 3 mM hippuryl-histidyl-leucine, 15 µL of 20 mg/mL *o*-phthaldialdehyde and 50 µL of the extract (0.4 mg/mL). The inhibitory activity was evaluated using an excitation wavelength of 360 nm and an emission wavelength of 485 nm as an end-point assay.

The acetylcholinesterase (AChE) inhibitory reaction consisted of 100 μL of 20 ng *Electrophorus electricus* AChE (1000 units/mg), 40 μL of 0.8 mM acetylthiocholine, 10 µL of 16 mM DTNB and 40 µL of the extract (5 mg/mL), while the butyrylcholinesterase (BChE) inhibitory reaction consisted of 100 µL of 0.5 µg/mL equine serum BChE (≥10 units/mg), 40 µL of 0.4 mM butyrylthiocholine, 10 µL of 16 mM DTNB and 40 µL of the extract (5 mg/mL). The inhibitory activity was visualized as a decline in enzyme kinetics at 412 nm. The β-secretase (BACE-1) inhibitory reaction was studied using a BACE-1 fluorescence resonance energy transfer (FRET) assay kit (Sigma-Aldrich, St. Louis, MO, USA) according to the manufacturer’s recommendations. The inhibitory activity of the extract (20 µL of 5 mg/mL) was evaluated using an excitation wavelength of 320 nm and an emission wavelength of 405 nm as an end-point assay. 

The anti-glycation reaction induced by D-glucose consisted of 50 µL of 20 mg/mL bovine serum albumin (BSA, ≥98.0% agarose gel electrophoresis) in 100 mM potassium phosphate buffer (pH 7.4) containing 0.02% (w/v) sodium azide, 25 µL of 1 M D-glucose and 25 µL of extract (2.52 mg/mL). For the anti-glycation reaction induced by methylglyoxal (MG), 25 µL of 4 mM MG was used instead of D-glucose. The reaction mixture was incubated at 37 °C for 2 weeks in the dark. The inhibitory activity was evaluated using an excitation wavelength of 330 nm and an emission wavelength of 410 nm as an end-point assay.

The percentage of enzyme inhibition using enzyme kinetics was calculated using the following equation:

$$\%\;\mathrm{inhibition}\;=\;\left(1-\frac{B-b}{A-a}\right)\;\times \;100,$$

where *A* is the initial velocity of the control reaction with enzyme (control), *a* is the initial velocity of the control reaction without enzyme (control blank), *B* is the initial velocity of the enzyme reaction with extract (sample) and *b* is the initial velocity of the reaction with extract but without enzyme (sample blank). The percentage of enzyme inhibition using an end-point assay and anti-glycation reaction was evaluated using the same equation but changing from initial velocity to absorbance at a particular wavelength.

### 4.5. Principal Component Analysis, Hierarchical Cluster Analysis, and Data Analysis

Principal component analysis (PCA) and hierarchical cluster analysis (HCA) of TPCs, antioxidant activities and enzymatic and non-enzymatic inhibitory activities of Thai vegetable extracts were performed using XLSTAT^®^ (version 2021, a trial version from Addinsoft Inc., New York, NY, USA) to create a biplot. 

All experiments were evaluated in triplicate (*n* = 3), with data expressed as mean ± standard deviation (SD). Statistical analysis was performed using the statistical package for the social sciences (version 18 for Windows, SPSS Inc., Chicago, IL, USA). Significant difference at *p* < 0.05 of more than two data was calculated using one–way analysis of variance (ANOVA), followed by Duncan’s multiple comparison test, while significantly difference at *p* < 0.05 of two data was calculated by Student’s *t*-test. 

## Figures and Tables

**Table 1:** Profiles and quantification of flavonoids in the plant samples.

Samples	Flavonoids (mg/100 g DW)						
	Quercetin	Kaempferol	Hesperidin	Luteolin	Apigenin	Delphinidin	Cyanidin
*A. cepa*	63.34 ± 1.01 ^b^	ND	ND	ND	ND	ND	13.35 ± 1.07
*A. fistulosum*	ND	47.97 ± 0.97 ^a^	ND	ND	ND	ND	ND
*A. sativum*	ND	ND	ND	ND	ND	ND	ND
*Ci. hystrix*	25.52 ± 0.36 ^c^	ND	453.47 ± 2.28	ND	ND	ND	ND
*Co. sativum*	166.16 ± 1.20 ^a^	4.44 ± 0.03 ^c^	ND	ND	ND	ND	ND
*Cy. citratus*	ND	ND	ND	4.57 ± 0.10	ND	ND	ND
*E. foetidum*	ND	22.09 ± 2.91 ^b^	ND	ND	ND	ND	ND
*P. tetragonolobus*	ND	ND	ND	ND	ND	15.77 ± 0.59	43.02 ± 0.87 *
*Se. pennata*	ND	ND	ND	ND	3.46 ± 0.10	ND	ND
*So. melongena*	ND	ND	ND	ND	ND	ND	ND

All data were expressed as mean ± standard deviation (SD) of triplicate experiments (*n* = 3). Small letters indicate significantly different contents of the same phenolic in different vegetable extracts (more than two samples) at *p* < 0.05 calculated by one–way analysis of variance (ANOVA) and Duncan’s multiple comparison test, while asterisk (*) indicates significantly different contents of the same phenolic in different vegetable extracts (two samples) at *p* < 0.05 calculated by Student’s *t*-test. ND: not detected.

**Table 2:** Profiles and quantification of phenolic acids in the plant samples.

Samples	Phenolic Acids (mg/100 g DW)				
	4-Hydroxybenzoic Acid	Vanillic Acid	Caffeic Acid	*p*-Coumaric Acid	Ferulic Acid
*A. cepa*	ND	ND	ND	ND	ND
*A. fistulosum*	ND	ND	ND	7.12 ± 0.16 ^b^	23.13 ± 0.12 ^b^
*A. sativum*	ND	ND	ND	ND	ND
*Ci. hystrix*	ND	ND	ND	ND	ND
*Co. sativum*	ND	3.73 ± 0.04	23.81 ± 0.50 ^c^	5.20 ± 0.06 ^c^	ND
*Cy. citratus*	ND	ND	15.65 ± 0.28 ^d^	68.13 ± 1.09 ^a^	123.34 ± 2.82 ^a^
*E. foetidum*	ND	ND	52.69 ± 8.33 ^b^	2.63 ± 0.24 ^e^	1.77 ± 0.01 ^c^
*P. tetragonolobus*	10.80 ± 2.79	15.71 ± 0.02 *	16.13 ± 5.37 ^d^	3.85 ± 0.44 ^d^	ND
*Se. pennata*	ND	ND	14.92 ± 0.09 ^d^	ND	ND
*So. melongena*	ND	ND	246.99 ± 10.55 ^a^	2.16 ± 0.04 ^e^	ND

All data were expressed as mean ± standard deviation (SD) of triplicate experiments (*n* = 3). Small letters indicate significantly different contents of the same phenolic in different vegetable extracts (more than two samples) at *p* < 0.05 calculated by one–way analysis of variance (ANOVA) and Duncan’s multiple comparison test, while asterisk (*) indicates significantly different contents of the same phenolic in different vegetable extracts (two samples) at *p* < 0.05 calculated by Student’s *t*-test. ND: not detected.

**Table 3:** Total phenolic contents and antioxidant activities of the ten vegetable extracts.

Sample	Total Phenolic Contents (mg GAE/g DW)	Antioxidant Activities (μmol TE/g DW)		
		DPPH Radical Scavenging Assay	FRAP Assay	ORAC Assay
*A. cepa*	3.76 ± 0.07 ^f^	5.90 ± 0.17 ^f^	5.10 ± 0.05 ^i^	15.12 ± 0.46 ^g^
*A. fistulosum*	5.91 ± 0.11 ^e^	6.14 ± 0.25 ^f^	18.32 ± 0.08 ^g^	223.83 ± 11.46 ^c^
*A. sativum*	1.23 ± 0.02 ^i^	1.77 ± 0.01 ^g^	3.25 ± 0.08 ^j^	80.14 ± 1.51 ^f^
*Ci. hystrix*	12.98 ± 0.15 ^b^	6.14 ± 0.06 ^f^	38.12 ± 0.43 ^e^	418.32 ± 0.77 ^a^
*Co. sativum*	2.68 ± 0.07 ^h^	9.12 ± 0.14 ^e^	16.46 ± 0.29 ^h^	87.46 ± 3.96 ^f^
*Cy. citratus*	9.49 ± 0.28 ^d^	6.23 ± 0.51 ^f^	43.21 ± 1.62 ^c^	119.91 ± 4.00 ^e^
*E. foetidum*	4.15 ± 0.08 ^g^	16.46 ± 0.35 ^c^	27.39 ± 1.65 ^f^	199.99 ± 8.64 ^d^
*P. tetragonolobus*	5.41 ± 0.20 ^e^	21.58 ± 0.88 ^b^	40.92 ± 1.38 ^d^	211.83 ± 3.89 ^cd^
*Se. pennata*	15.33 ± 0.64 ^a^	33.06 ± 1.07 ^a^	62.33 ± 1.97 ^a^	266.11 ± 9.93 ^b^
*So. melongena*	10.07 ± 0.21 ^c^	12.07 ± 0.36 ^d^	57.15 ± 0.32 ^b^	415.92 ± 18.78 ^a^

All data were expressed as mean ± standard deviation (SD) of triplicate experiments (*n* = 3). Small letters indicate significantly different values of the same assay in different vegetable extracts at *p* < 0.05 calculated by one-way analysis of variance (ANOVA) and Duncan’s multiple comparison test. GAE: gallic acid equivalent; TE: Trolox equivalent; DW: dry weight; DPPH: 2,2-diphenyl-1-picrylhydrazyl; FRAP: ferric reducing antioxidant power; ORAC: oxygen radical absorbance capacity.

**Table 4:** Enzyme inhibitory activities of the ten vegetable extracts.

Samples	Enzyme Inhibitory Activities (%Inhibition)						
	Lipase	α-Amylase	α-Glucosidase	ACE	AChE	BChE	BACE-1
*A. cepa*	12.86±0.37 ^h^	19.16±0.38 ^d^	8.09 ± 0.38 ^h^	91.31 ± 1.43 ^a^	8.32 ± 0.30 ^g^	8.44 ± 0.07 ^g^	35.22 ± 2.11 ^b^
*A. fistulosum*	33.76±0.21 ^f^	15.02±0.41 ^e^	40.82 ± 0.66 ^b^	60.01 ± 1.79 ^e^	21.31 ± 0.66 ^cd^	17.34 ± 1.16 ^f^	39.14 ± 1.04 ^a^
*A. sativum*	46.44±1.43 ^c^	4.11±0.30 ^i^	ND	76.15 ± 1.39 ^c^	ND	8.55 ± 0.20 ^g^	ND
*Ci. hystrix*	61.16±1.33 ^a^	26.62±0.32 ^c^	33.76 ± 1.60 ^d^	91.71 ± 2.11 ^a^	29.09 ± 1.07 ^b^	52.61 ± 1.60 ^a^	24.76 ± 1.44 ^e^
*Co. sativum*	55.76±1.40 ^b^	58.43±0.56 ^a^	18.55 ± 0.57 ^g^	70.66 ± 2.34 ^d^	22.34 ± 0.74 ^c^	40.09 ± 1.06 ^b^	28.00 ± 0.49 ^d^
*Cy. citratus*	39.15±2.29 ^e^	4.93±0.01 ^hi^	29.57 ± 0.44 ^f^	68.80 ± 0.74 ^d^	12.40 ± 0.70 ^f^	22.70 ± 0.35 ^e^	12.46 ± 1.28 ^h^
*E. foetidum*	43.28±0.85 ^d^	31.22±1.21 ^b^	38.36 ± 0.96 ^c^	68.53 ± 0.88 ^d^	58.57 ± 0.51 ^a^	32.85 ± 0.47 ^c^	20.39 ± 1.15 ^f^
*P. tetragonolobus*	39.15±2.29 ^e^	10.51±0.96 ^f^	64.03 ± 1.30 ^a^	76.80 ± 3.58 ^c^	28.18 ± 1.30 ^b^	23.63 ± 1.24 ^e^	30.98 ± 1.41 ^c^
*Se. pennata*	44.95±0.66 ^cd^	5.16±0.09 ^h^	19.78 ± 0.54 ^g^	87.29 ± 1.28 ^b^	20.22 ± 1.13 ^d^	28.12 ± 1.20 ^d^	36.39 ± 0.79 ^b^
*So. melongena*	20.85±0.52 ^g^	7.66±0.33 ^g^	32.03 ± 1.27 ^e^	50.35 ± 1.17 ^f^	16.59 ± 0.13 ^e^	3.65 ± 0.15 ^h^	15.53 ± 0.21 ^g^

All data are expressed as mean ± standard deviation (SD) of triplicate experiments (*n* = 3). Small letters indicate significantly different inhibitory activities of the same enzyme assay in different vegetable extracts at *p* < 0.05 calculated by the one-way analysis of variance (ANOVA) and Duncan’s multiple comparison test. The final concentration of the extracts in all enzymatic assays was 1 mg/mL, with the exception of ACE inhibitory assay, which was 0.2 mg/mL. ACE: angiotensin-converting enzyme; AChE: acetylcholinesterase; BChE: butyrylcholinesterase; BACE-1: β-secretase; ND: not detected.

**Table 5:** Non-enzyme inhibitory activities of the ten vegetable extracts.

Samples	Anti-Glycation Reaction (%Inhibition)	
	D-Glucose Induction	Methylglyoxal Induction
*A. cepa*	21.26 ± 1.78 ^f^	5.09 ± 0.04 ^i^
*A. fistulosum*	40.06 ± 1.11 ^e^	29.83 ± 2.03 ^h^
*A. sativum*	10.63 ± 0.45 ^g^	5.17 ± 1.18 ^i^
*Ci. hystrix*	52.30 ± 1.85 ^d^	63.79 ± 0.48 ^c^
*Co. sativum*	65.61 ± 0.73 ^b^	57.63 ± 0.65 ^b^
*Cy. citratus*	39.82 ± 0.65 ^e^	39.85 ± 1.73 ^g^
*E. foetidum*	61.40 ± 1.61 ^c^	50.53 ± 0.24 ^e^
*P. tetragonolobus*	50.96 ± 0.52 ^d^	53.13 ± 1.24 ^d^
*Se. pennata*	74.55 ± 0.71 ^a^	81.54 ± 2.68 ^a^
*So. melongena*	51.87 ± 1.06 ^d^	47.21 ± 0.69 ^f^

All data are expressed as mean ± standard deviation (SD) of triplicate experiments (*n* = 3). Small letters indicate significant difference in anti-glycation reactions induced with the same inducer (D-glucose or methylglyoxal) of different vegetable extracts at *p* < 0.05 calculated by one-way analysis of variance (ANOVA) and Duncan’s multiple comparison test. Final concentration of the extracts in the assays was 0.63 mg/mL.

## Data Availability

Data is contained within this article and supplementary material.

## Supplementary Materials

The following are available online at https://www.mdpi.com/article/10.3390/plants10081563/s1, **Table S1**. The cultivation area, voucher specimen, harvesting time and physical appearance of the edible part of the samples; **Table S2**. The percentage of edible part and colors of fresh samples. The color value was determined using a ColorFlex EZ spectrophotometer and expressed as CIELAB units (L* represents dark (0) to white (100) colors, a* represents green (−) to red (+) colors, and b* represents blue (−) to yellow (+) colors); **Table S3.** The validation parameters of phenolics detection using HPLC analysis; **Figure S1**. High-performance liquid chromatograms showing retention time (*Rt*) of (A) authentic standards including gallic acid (4.8 min), 4-hydroxybenzoic acid (12.1 min), chlorogenic acid (13.3 min), vanillic acid (14.6 min), caffeic acid (15.3 min), syringic acid (16.5 min), *p*-coumaric acid (22.4 min), ferulic acid (22.5 min) and sinapic acid (26.5 min), (B.) *A. cepa*, (C.) *A. fistulosum*, (D.) *A. sativum*, (E.) *Ci. hystrix*, (F.) *Co. sativum*, (G.) *Cy. citratus*, (H.) *E. foetidum*, (I.) *P. tetragonolobus*, (J.) *Se. pennata*, and (K.) *So. melongena* detected at 280 nm; **Figure S2**. High-performance liquid chromatograms showing retention time (*Rt*) of (A) authentic standards including chlorogenic acid (13.3 min), caffeic acid (15.3 min), *p*-coumaric acid (22.2 min), ferulic acid (25.4 min), sinapic acid (26.5 min), myricetin (37.9 min), quercetin (43.1 min), luteolin (43.7 min), hesperitin (45.2 min), kaempferol (46.2 min), apigenin (46.5 min) and isorhamnetin (47.1 min), (B.) *A. cepa*, (C.) *A. fistulosum*, (D.) *A. sativum*, (E.) *Ci. hystrix*, (F.) *Co. sativum*, (G.) *Cy. citratus*, (H.) *E. foetidum*, (I.) *P. tetragonolobus*, (J.) *Se. pennata*, and (K.) *So. melongena* detected at 338 nm; **Figure S3**. High-performance liquid chromatograms showing retention time (*Rt*) of (A) authentic standards including delphinidin (29.0 min) and cyanidin (35.3 min), (B.) *A. cepa*, (C.) *A. fistulosum*, (D.) *A. sativum*, (E.) *Ci. hystrix*, (F.) *Co. sativum*, (G.) *Cy. citratus*, (H.) *E. foetidum*, (I.) *P. tetragonolobus*, (J.) *Se. pennata*, and (K.) *So. melongena* detected at 524 nm.

## Abbreviations

ACE	Angiotensin-converting enzyme
AChE	Acetylcholinesterase
AD	Alzheimer’s disease
BACE-1	β-secretase
BChE	Butyrylcholinesterase
DPPH	2,2-diphenyl-1-picrylhydrazyl
DW	Dry weight
FRAP	Ferric reducing antioxidant power
HCA	Hierarchical cluster analysis
MG	Methylglyoxal
NCDs	Non-communicable diseases
ORAC	Oxygen radical absorbance capacity
PCA	Principal component analysis
TPCs	Total phenolic contents

## References

[1] World Health Organization (WHO) (2016). Global Status Report on Noncommunicable Disease 2014.

[2] World Health Organization (WHO) (2005). Fruit and Vegetables for Health: Report of the Joint FAO/WHO Workshop on Fruit and Vegetables for Health, Kobe, Japan, 1–3 September 2004.

[3] Borgi L., Muraki I., Satija A., Willett W.C., Rimm E.B., Forman J.P. (2016). Fruit and Vegetable Consumption and the Incidence of Hypertension in Three Prospective Cohort Studies. Hypertension.

[4] Chiavaroli L., Viguiliouk E., Nishi S.K., Blanco Mejia S., Rahelic D., Kahleova H., Salas-Salvado J., Kendall C.W., Sievenpiper J.L. (2019). DASH Dietary Pattern and Cardiometabolic Outcomes: An Umbrella Review of Systematic Reviews and Meta-Analyses. Nutrients.

[5] Agarwal P., Holland T.M., Wang Y., Bennett D.A., Morris M.C. (2019). Association of Strawberries and Anthocyanidin Intake with Alzheimer’s Dementia Risk. Nutrients.

[6] Holland T.M., Agarwal P., Wang Y., Leurgans S.E., Bennett D.A., Booth S.L., Morris M.C. (2020). Dietary flavonols and risk of Alzheimer dementia. Neurology.

[7] Thakur M., Singh K., Khedkar R., Prakash B. (2020). Phytochemicals: Extraction process, safety assessment, toxicological evaluations, and regulatory issues. Functional and Preservative Properties of Phytochemicals.

[8] Halliwell B., Murcia M.A., Chirico S., Aruoma O.I. (1995). Free radicals and antioxidants in food and *in vivo*: What they do and how they work. Crit. Rev. Food Sci. Nutr..

[9] Zhu F., Du B., Xu B. (2018). Anti-inflammatory effects of phytochemicals from fruits, vegetables, and food legumes: A review. Crit. Rev. Food Sci. Nutr..

[10] Chen H., Liu R.H. (2018). Potential Mechanisms of Action of Dietary Phytochemicals for Cancer Prevention by Targeting Cellular Signaling Transduction Pathways. J. Agric. Food Chem..

[11] Hinkaew J., Aursalung A., Sahasakul Y., Tangsuphoom N., Suttisansanee U. (2021). A Comparison of the Nutritional and Biochemical Quality of Date Palm Fruits Obtained Using Different Planting Techniques. Molecules.

[12] Temviriyanukul P., Sritalahareuthai V., Jom K.N., Jongruaysup B., Tabtimsri S., Pruesapan K., Thangsiri S., Inthachat W., Siriwan D., Charoenkiatkul S. (2020). Comparison of Phytochemicals, Antioxidant, and In Vitro Anti-Alzheimer Properties of Twenty-Seven Morus spp. Cultivated in Thailand. Molecules.

[13] Wannasaksri W., On-Nom N., Chupeerach C., Temviriyanukul P., Charoenkiatkul S., Suttisansanee U. (2021). In Vitro Phytotherapeutic Properties of Aqueous Extracted Adenia viridiflora Craib. towards Civilization Diseases. Molecules.

[14] Chaudhuri J., Bains Y., Guha S., Kahn A., Hall D., Bose N., Gugliucci A., Kapahi P. (2018). The Role of Advanced Glycation End Products in Aging and Metabolic Diseases: Bridging Association and Causality. Cell Metab..

[15] Nagai R., Shirakawa J., Ohno R., Moroishi N., Nagai M. (2014). Inhibition of AGEs formation by natural products. Amino Acids.

[16] Verma T., Sinha M., Bansal N., Yadav S.R., Shah K.A.-O., Chauhan N.A.-O. (2021). Plants Used as Antihypertensive. Nat. Prod. Bioprospect..

[17] Geck M.S., Cristians S., Berger-González M., Casu L., Heinrich M., Leonti M. (2020). Traditional Herbal Medicine in Mesoamerica: Toward Its Evidence Base for Improving Universal Health Coverage. Front. Pharmacol..

[18] Sanlier N., Gencer F. (2020). Role of spices in the treatment of diabetes mellitus: A minireview. Trends Food Sci. Technol..

[19] Thomas P.S., Essien E.E., Ntuk S.J., Choudhary M.I. (2017). *Eryngium foetidum* L. Essential Oils: Chemical Composition and Antioxidant Capacity. Medicines.

[20] Wu T.T., Tsai C.W., Yao H.T., Lii C.K., Chen H.W., Wu Y.L., Chen P.Y., Liu K.L. (2010). Suppressive effects of extracts from the aerial part of *Coriandrum sativum* L. on LPS-induced inflammatory responses in murine RAW 264.7 macrophages. J. Sci. Food Agric..

[21] Sung Y.Y., Kim D.S., Kim S.H., Kim H.K. (2018). Aqueous and ethanolic extracts of welsh onion, Allium fistulosum, attenuate high-fat diet-induced obesity. BMC Complement. Altern. Med..

[22] Ried K. (2020). Garlic lowers blood pressure in hypertensive subjects, improves arterial stiffness and gut microbiota: A review and meta-analysis. Exp. Ther. Med..

[23] Liu T.T., Liu X.T., Chen Q.X., Shi Y. (2020). Lipase Inhibitors for Obesity: A Review. Biomed. Pharm..

[24] Tundis R., Loizzo M.R., Menichini F. (2010). Natural products as alpha-amylase and alpha-glucosidase inhibitors and their hypoglycaemic potential in the treatment of diabetes: An update. Mini Rev. Med. Chem..

[25] Breijyeh Z., Karaman R. (2020). Comprehensive Review on Alzheimer’s Disease: Causes and Treatment. Molecules.

[26] Vlassara H. (2005). Advanced glycation in health and disease: Role of the modern environment. Ann. N. Y. Acad. Sci..

[27] Patel S., Sihmar S., Jatain A. (2015). A Study of Hierarchical Clustering Algorithms. Proceedings of the 2015 2nd International Conference on Computing for Sustainable Global Development (INDIACom).

[28] Aryal S., Baniya M.K., Danekhu K., Kunwar P., Gurung R., Koirala N. (2019). Total Phenolic Content, Flavonoid Content and Antioxidant Potential of Wild Vegetables from Western Nepal. Plants.

[29] Nanasombat S., Teckchuen N. (2009). Antimicrobial, antioxidant and anticancer activities of Thai local vegetables. J. Med. Plants Res..

[30] Ramli S., Bunrathep S., Tansaringkarn T., Ruangrungsi N. (2018). Screening for Free Radical Scavenging Activity from Ethanolic Extract of Mimosaceous Plants Endemic to Thailand. J. Health Res..

[31] Chokthaweepanich H., Sriwicha S., Auvuchanon A., Supapvanich S. (2021). Phytochemical Screening and Fruit Quality of Commercial Eggplants. Cast.

[32] Wijaya Y.A., Widyadinata D., Irawaty W., Ayucitra A. (2017). Fractionation of Phenolic Compounds from Kaffir Lime (*Citrus Hystrix*) Peel Extract and Evaluation of Antioxidant Activity. Reaktor.

[33] Kawaguchi K., Mizuno T., Aida K., Uchino K. (1997). Hesperidin as an Inhibitor of Lipases from Porcine Pancreas and Pseudomonas. Biosci. Biotechnol. Biochem..

[34] Martinez-Gonzalez A.I., Alvarez-Parrilla E., Díaz-Sánchez Á.G., de la Rosa L.A., Núñez-Gastélum J.A., Vazquez-Flores A.A., Gonzalez-Aguilar G.A. (2017). In vitro Inhibition of Pancreatic Lipase by Polyphenols: A Kinetic, Fluorescence Spectroscopy and Molecular Docking Study. Food Technol. Biotechnol..

[35] Bustos A.-S., Håkansson A., Linares-Pastén J.A., Penarrieta J.M., Nilsson L. (2018). Interaction Between Phenolic Compounds and Lipase: The Influence of Solubility and Presence of Particles in the IC50 Value. J. Food Sci..

[36] Aligita W., Susilawati E., Septiani H., Atsil R. (2018). Antidiabetic Activity of Coriander (*Coriandrum sativum* L.) Leaves’ Ethanolic Extract. Int. J. Pharm. Phytopharm. Res..

[37] Narkhede M.B. (2012). Evaluation of Alpha Amylase Inhibitory Potential of Four Traditional Culinary Leaves. Asian J. Pharm. Clin. Res..

[38] Oboh G., Agunloye O.M., Adefegha S.A., Akinyemi A.J., Ademiluyi A.O. (2015). Caffeic and chlorogenic acids inhibit key enzymes linked to type 2 diabetes (in vitro): A comparative study. J. Basic Clin. Physiol. Pharmacol..

[39] Shori A.B. (2020). Proteolytic activity, antioxidant, and α-Amylase inhibitory activity of yogurt enriched with coriander and cumin seeds. LWT Food Sci. Technol..

[40] Mechchate H., Es-Safi I., Amaghnouje A., Boukhira S., Alotaibi A.A., Al-Zharani M., Nasr A.F., Noman M.O., Conte R., Amal E.H. (2021). Antioxidant, Anti-Inflammatory and Antidiabetic Proprieties of LC-MS/MS Identified Polyphenols from Coriander Seeds. Molecules.

[41] Hajlaoui H., Arraouadi S., Noumi E., Aouadi K., Adnan M., Khan M.A., Kadri A., Snoussi M. (2021). Antimicrobial, Antioxidant, Anti-Acetylcholinesterase, Antidiabetic, and Pharmacokinetic Properties of *Carum carvi* L. and *Coriandrum sativum* L. Essential Oils Alone and in Combination. Molecules.

[42] Promyos N., Temviriyanukul P., Suttisansanee U. (2020). Investigation of Anthocyanidins and Anthocyanins for Targeting alpha-Glucosidase in Diabetes Mellitus. Prev. Nutr. Food Sci..

[43] Hossain U., Das A.K., Ghosh S., Sil P.C. (2020). An overview on the role of bioactive α-glucosidase inhibitors in ameliorating diabetic complications. Food Chem. Toxicol..

[44] Tadera K., Minami Y., Takamatsu K., Matsuoka T. (2006). Inhibition of alpha-glucosidase and alpha-amylase by flavonoids. J. Nutr. Sci. Vitam..

[45] Brüll V., Burak C., Stoffel-Wagner B., Wolffram S., Nickenig G., Müller C., Langguth P., Alteheld B., Fimmers R., Naaf S. (2015). Effects of a quercetin-rich onion skin extract on 24 h ambulatory blood pressure and endothelial function in overweight-to-obese patients with (pre-)hypertension: A randomised double-blinded placebo-controlled cross-over trial. Br. J. Nutr..

[46] Guerrero L., Castillo J., Quiñones M., Garcia-Vallvé S., Arola L., Pujadas G., Muguerza B. (2012). Inhibition of angiotensin-converting enzyme activity by flavonoids: Structure-activity relationship studies. PLoS ONE.

[47] Bhullar K.S., Lassalle-Claux G., Touaibia M., Rupasinghe H.P.V. (2014). Antihypertensive effect of caffeic acid and its analogs through dual renin–angiotensin–aldosterone system inhibition. Eur. J. Pharmacol..

[48] Kean R.J., Lamport D.J., Dodd G.F., Freeman J.E., Williams C.M., Ellis J.A., Butler L.T., Spencer J.P. (2015). Chronic consumption of flavanone-rich orange juice is associated with cognitive benefits: An 8-wk, randomized, double-blind, placebo-controlled trial in healthy older adults. Am. J. Clin. Nutr..

[49] Wang Y., Wang Y., Li J., Hua L., Han B., Zhang Y., Yang X., Zeng Z., Bai H., Yin H. (2016). Effects of caffeic acid on learning deficits in a model of Alzheimer’s disease. Int. J. Mol. Med..

[50] Lee S., Youn K., Lim G., Lee J., Jun M. (2018). In Silico Docking and *In Vitro* Approaches towards BACE1 and Cholinesterases Inhibitory Effect of Citrus Flavanones. Molecules.

[51] Oboh G., Agunloye O.M., Akinyemi A.J., Ademiluyi A.O., Adefegha S.A. (2013). Comparative study on the inhibitory effect of caffeic and chlorogenic acids on key enzymes linked to Alzheimer’s disease and some pro-oxidant induced oxidative stress in rats’ brain-in vitro. Neurochem. Res..

[52] Shimmyo Y., Kihara T., Akaike A., Niidome T., Sugimoto H. (2008). Flavonols and flavones as BACE-1 inhibitors: Structure-activity relationship in cell-free, cell-based and in silico studies reveal novel pharmacophore features. Biochim. Biophys. Acta.

[53] Hunt J.V., Wolff S.P. (1991). Oxidative glycation and free radical production: A causal mechanism of diabetic complications. Free Radic. Res. Commun..

[54] Ronsisvalle S., Panarello F., Longhitano G., Siciliano E.A., Montenegro L., Panico A. (2020). Natural Flavones and Flavonols: Relationships among Antioxidant Activity, Glycation, and Metalloproteinase Inhibition. Cosmetics.

[55] Sasaki K., Chiba S., Yoshizaki F. (2014). Effect of natural flavonoids, stilbenes and caffeic acid oligomers on protein glycation. Biomed. Rep..

[56] Wang R., Lu X., Sun Q., Gao J., Ma L., Huang J. (2020). Novel ACE Inhibitory Peptides Derived from Simulated Gastrointestinal Digestion *in Vitro* of Sesame (*Sesamum indicum* L.) Protein and Molecular Docking Study. Int. J. Mol. Sci..

[57] Srinuanchai W., Nooin R., Jarussophon S., Kasemwong K., Nuchuchua O. (2019). Determination of gymnemic acid level in *Gymnema inodorum* leaves using multiple reaction monitoring mass spectrometry. J. Chem. Metrol..

[58] Sripum C., Kukreja R.K., Charoenkiatkul S., Kriengsinyos W., Suttisansanee U. (2016). The effect of storage conditions on antioxidant activities and total phenolic contents of parboiled germinated brown rice (Khao Dok Mali 105). Int. Food Res. J..

[59] Sripum C., Kukreja R.K., Charoenkiatkul S., Kriengsinyos W., Suttisansanee U. (2017). The effect of extraction conditions on antioxidant activities and total phenolic contents of different processed Thai Jasmine rice. Int. Food Res. J..

[60] Vinson J.A., Howard T.B. (1996). Inhibition of protein glycation and advanced glycation end products by ascorbic acid and other vitamins and nutrients. J. Nutr. Biochem..

