# Supplementary material for: Phytochemicals and In Vitro Bioactivities of Aqueous Ethanolic Extracts from Common Vegetables in Thai Food
Source: Plants (Basel). 2021 Jul 29;10(8):1563. doi: 10.3390/plants10081563 (PMC8400534; doi:10.3390/plants10081563)
Supplement: Supplementary file 1 [file plants-10-01563-s001.zip › plants-1311981-supplementary.pdf]

Supplementary materials

Phytochemicals and *In vitro* Bioactivities of Aqueous Ethanolic Extracts from Common Vegetables in Thai Food

Uthaiwan Suttisansanee [1,2], Parunya Thiyajai [1,2], Parisut Chalermchaiwat [3], Khanitha Wongwathanarat [4], Kanchana Pruesapan [5], Somsri Charoenkiatkul [1] and Piya Temviriyanukul [1,2,*]

[1] Institute of Nutrition, Mahidol University, Salaya, Phuttamonthon, Nakhon Pathom 73170, Thailand; uthaiwan.sut@mahidol.ac.th (U.S.); parunya.thy@mahidol.ac.th (P.Th.); somsri.chr@mahidol.ac.th (S.C.); piya.tem@mahidol.ac.th (P.T.)
[2] Food and Nutrition Academic and Research Cluster, Institute of Nutrition, Mahidol University, Salaya, Phuttamonthon, Nakhon Pathom 73170, Thailand
[3] Food and Nutrition Program, Department of Home Economics, Faculty of Agriculture, Kasetsart University Bangkok 10900, Thailand; fagrpsch@ku.ac.th (P.C.)
[4] Biotechnology Research and Development Office, Department of Agriculture, Ministry of Agriculture and Cooperatives, Bangkok 10900, Thailand; kwongwath@yahoo.com (K.W.)
[5] Plant Varieties Protection Division, Department of Agriculture, Ministry of Agriculture and Cooperatives, Bangkok 10900, Thailand; kpruesapan@gmail.com (K.P.)
* Correspondence: piya.tem@mahidol.ac.th; Tel.: +66-(0)-2800-2380 (ext. 326)

Supplementary Table S1:

The cultivation area, voucher specimen, harvesting time and physical appearance of the edible part of the samples.

**Scientific name and cultivation area**	**Voucher specimen and harvesting time**	**Physical appearance of the edible part**
Allium cepa Aggregatum Group from Nong Phai subdistrict, Mueang Si Sa Ket district, Si Sa Ket, Thailand	BK069175-7/ 80 days after planting	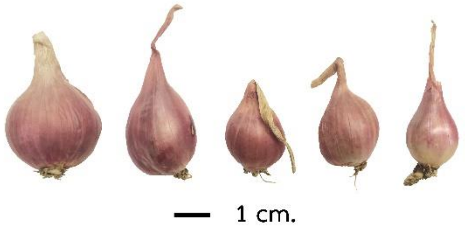 Edible part: bulbs
Allium fistulosum L. from Chai Chumphon subdistrict, Laplae district, Uttaradit, Thailand	BK069094/ 45 days after seeding	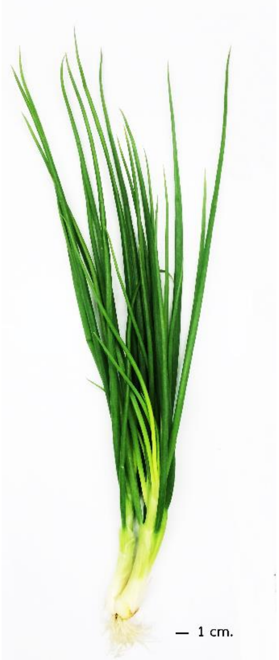 Edible part: leaves
Allium sativum L. from Khayung subdistrict, Uthumphon Phisai district, Si Sa Ket, Thailand	BK069066/ 90 days after planting	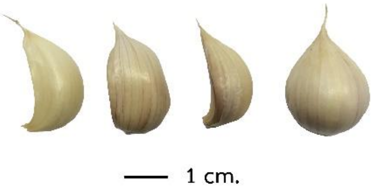 Edible parts: bulbs

Supplementary Table S1 (Cont.):

The cultivation area, voucher specimen, harvesting time and physical appearance of the edible part of the samples.

Scientific name and cultivation area	Voucher specimen and harvesting time	Physical appearance and edible part
Citrus hystrix DC.<br>from Chorakhe Phueak subdistrict, Dan Makham Tia district, Kanchanaburi, Thailand	BK069139-42/<br>150 days after flowering	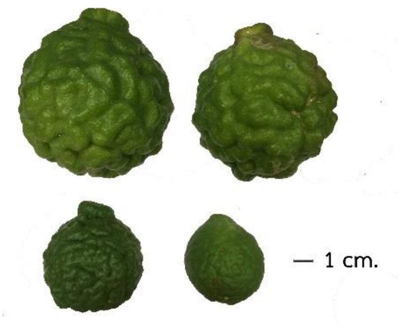<br><br>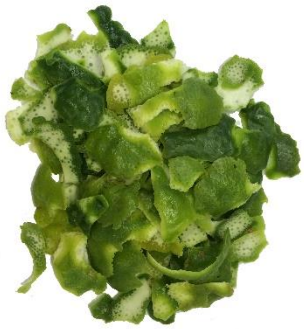<br>Edible part: fruit peel
Coriandrum sativum L.<br>from Ban Hong subdistrict, Ban Hong district, Lamphun, Thailand	BK069108-10/<br>45 days after seeding	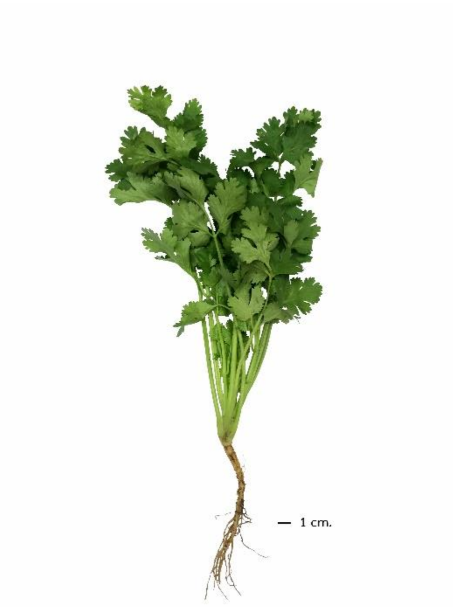<br><br>Edible part: leaves

Supplementary Table S1 (Cont.):

The cultivation area, voucher specimen, harvesting time and physical appearance of the edible part of the samples.

Scientific name and cultivation area	Voucher specimen and harvesting time	Physical appearance of the edible part
Cymbopogon citratus (DC.) Stapf from Thoet Thai subdistrict, Mae Fa Luang district, Chiang Rai, Thailand	BK069099-101/ 180 days after planting	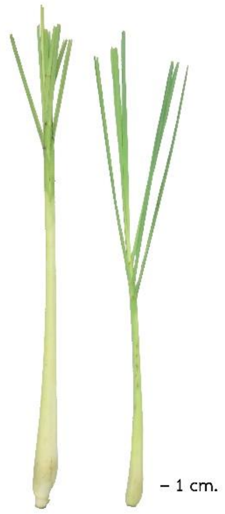 Edible part: stalk
Eryngium foetidum L. from Ban Hong subdistrict, Ban Hong district, Lamphun, Thailand	BK069114-6/ 120 days after seeding	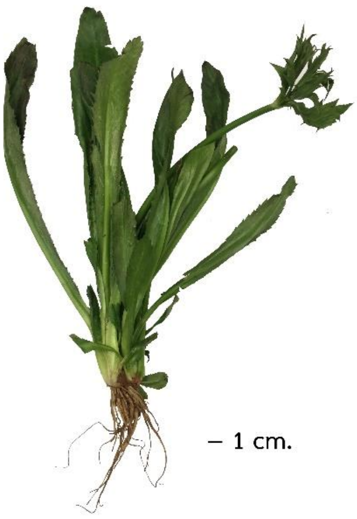 Edible part: leaves

Supplementary Table S1 (Cont.):

The cultivation area, voucher specimen, harvesting time and physical appearance of the edible part of the samples.

Scientific name and cultivation area	Voucher specimen and harvesting time	Physical appearance of the edible part
Psophocarpus tetragonolobus (L.) DC. From Nong Ya Lat subdistrict, Kantharalak district, Si Sa Ket, Thailand	BK069105-7/ 10-14 days after flowering	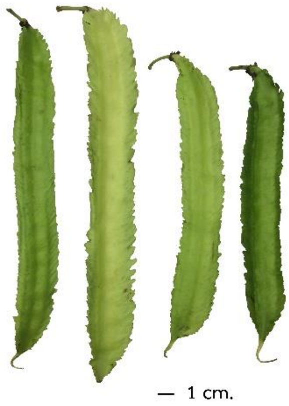 Edible part: whole fruits
Senegalia pennata subsp. *insuavis* (Lace) Maslin, Seigler & Ebinger from Phai Luang subdistrict, Taphan Hin district, Phichit, Thailand	BK069089-91/ 10-15 days after shooting	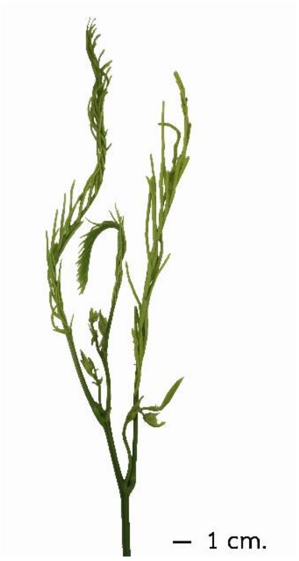 Edible part: young leaves
Solanum melongena 'Kermit' from Thung Thong subdistrict, Tha Muang district, Kanchanaburi, Thailand	BK069148-50/ 15-20 days after flowering	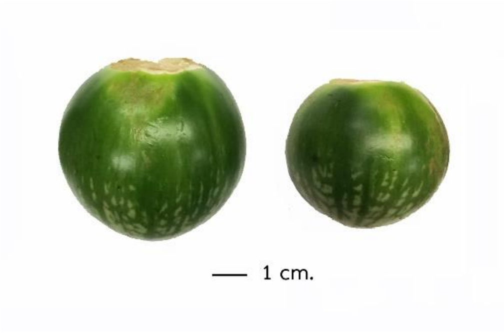 Edible part: whole fruits

Supplementary Table S2:

The percentage of edible part and color value of fresh samples. The color value was determined using a ColorFlex EZ spectrophotometer and expressed as CIELAB units (L* represents dark (0) to white (100) colors, a* represents green (-) to red (+) colors, and b* represents blue (-) to yellow (+) colors).

Samples	Color value			% of edible part
	L*	a*	b*	
Allium cepa Aggregatum Group	38.13 ± 4.38	6.10 ± 1.75	(-1.19) ± 1.26	78.70 ± 5.47
Allium fistulosum L.	30.99 ± 0.61	(-4.38) ± 0.14	7.57 ± 0.30	94.94 ± 0.42
Allium sativum L.	46.36 ± 0.64	(-0.62) ± 0.15	10.23 ± 0.46	83.41 ± 1.21
Citrus hystrix DC.	29.62 ± 0.96	(-4.14) ± 0.60	10.99 ± 1.60	27.63 ± 0.08
Coriandrum sativum L.	30.04 ± 1.25	(-4.95) ± 0.24	11.35 ± 0.53	82.11 ± 3.26
Cymbopogon citratus (DC.) Stapf	24.90 ± 0.52	0.90 ± 0.11	9.88 ± 0.08	43.58 ± 4.95
Eryngium foetidum L.	30.63 ± 0.36	(-5.02) ± 0.07	11.15 ± 0.67	58.29 ± 7.94
Psophocarpus tetragonolobus (L.) DC.	34.15 ± 0.76	(-4.80) ± 0.14	15.45 ± 0.28	94.28 ± 0.10
Senegalia pennata subsp. *insuavis* (Lace) Maslin, Seigler & Ebinger	31.28 ± 0.01	(-11.4) ± 0.01	31.05 ± 0.05	35.69 ± 1.56
Solanum melongena 'Kermit'	32.80 ± 3.03	(-4.45) ± 0.18	10.01 ± 1.96	92.06 ± 0.49

Supplementary Table S3:

The validation parameters of phenolics detection using HPLC analysis.

Standards	Linear range (μg/mL)	Linear regression equation	Correlation coefficient (R^2)	LOD (μg/mL)	LOQ (μg/mL)	RSD (%)
**Phenolic acids**						
Gallic acid	0.78-200.00	y = 38.814x + 43.836	0.9998	2.66	8.07	1.37
4-Hydroxybenzoic acid	0.39-100.00	y = 54.999x - 11.469	0.9994	0.32	0.98	1.86
Caffeic acid	0.39-100.00	y = 76.614x + 15.797	0.9998	0.59	1.78	1.70
Chlorogenic acid	0.39-100.00	y = 45.107x - 8.1659	1.0000	0.03	0.10	1.59
Ferulic acid	0.56-142.86	y = 42.477x - 16.212	0.9990	0.49	1.48	1.58
p-Coumaric acid	0.56-142.86	y = 44.809x - 22.611	0.9991	0.57	1.73	1.97
Sinapic acid	0.39-100.00	y = 63.807x + 10.841	1.0000	0.22	0.68	1.01
Syringic acid	0.65-166.67	y = 37.286x - 24.557	0.9990	1.12	3.41	1.63
Vanillic acid	0.65-333.33	y = 29.028x + 30.817	0.9999	2.19	6.64	1.99
**Flavonoids**						
Apigenin	0.39-200.00	y = 40.778x + 34.994	0.9999	2.22	6.73	0.44
Hesperidin	0.65-333.33	y = 24.424x + 76.057	0.9990	2.56	7.77	0.64
Kaempferol	0.65-166.67	y = 47.952x + 43.855	0.9999	0.14	0.42	0.12
Luteolin	0.65-166.67	y = 44.717x + 28.899	0.9999	1.19	3.62	0.52
Myricetin	0.65-166.67	y = 43.048x + 16.879	0.9999	0.37	1.12	0.32
Naringenin	0.65-333.33	y = 30.205x + 46.292	0.9999	0.46	1.38	0.45
Quercetin	0.65-166.67	y = 46.975x + 38.753	0.9999	0.19	0.58	0.10
Isorhamnetin	1.30-333.33	y = 16.302x + 46.483	0.9992	0.45	1.37	0.39

LOD : limit of quantitation; LOD : limit of detection; RSD : and relative standard deviation

Supplementary Figure S1:

High-performance liquid chromatograms showing retention time (*Rt*) of (A) authentic standards including gallic acid (4.8 min), 4-hydroxybenzoic acid (12.1 min), chlorogenic acid (13.3 min), vanillic acid (14.6 min), caffeic acid (15.3 min), syringic acid (16.5 min), *p*-coumaric acid (22.4 min), ferulic acid (22.5 min) and sinapic acid (26.5 min), (B.) *A. cepa*, (C.) *A. fistulosum*, (D.) *A. sativum*, (E.) *Ci. hystrix*, (F.) *Co. sativum*, (G.) *Cy. citratus*, (H.) *E. foetidum*, (I.) *P. tetragonolobus*, (J.) *Se. pennata*, and (K.) *So. melongena* detected at 280 nm.

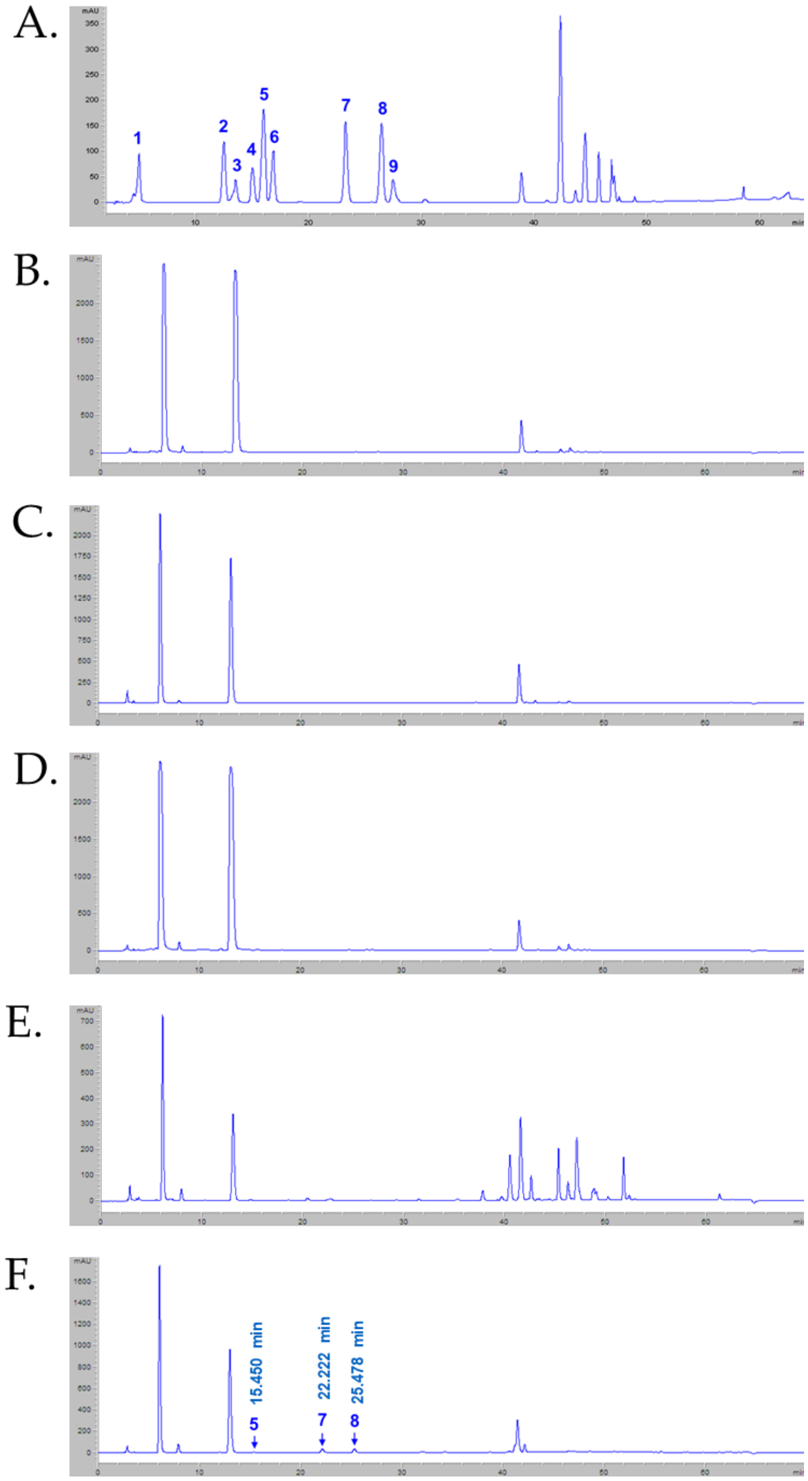

1 = gallic acid (4.8 min)
2 = 4-hydroxybenzoic acid (12.1 min)
3 = chlorogenic acid (13.3 min)
4 = vanillic acid (14.6 min)
5 = caffeic acid (15.3 min)
6 = syringic acid (16.5 min)
7 = *p*-coumaric acid (22.4 min)
8 = ferulic acid (22.5 min)
9 = sinapic acid (26.5)

Supplementary Figure S1 (Cont.):

High-performance liquid chromatograms showing retention time (*Rt*) of (A) authentic standards including gallic acid (4.8 min), 4-hydroxybenzoic acid (12.1 min), chlorogenic acid (13.3 min), vanillic acid (14.6 min), caffeic acid (15.3 min), syringic acid (16.5 min), *p*-coumaric acid (22.4 min), ferulic acid (22.5 min) and sinapic acid (26.5 min), (B.) *A. cepa*, (C.) *A. fistulosum*, (D.) *A. sativum*, (E.) *Ci. hystrix*, (F.) *Co. sativum*, (G.) *Cy. citratus*, (H.) *E. foetidum*, (I.) *P. tetragonolobus*, (J.) *Se. pennata*, and (K.) *So. melongena* detected at 280 nm.

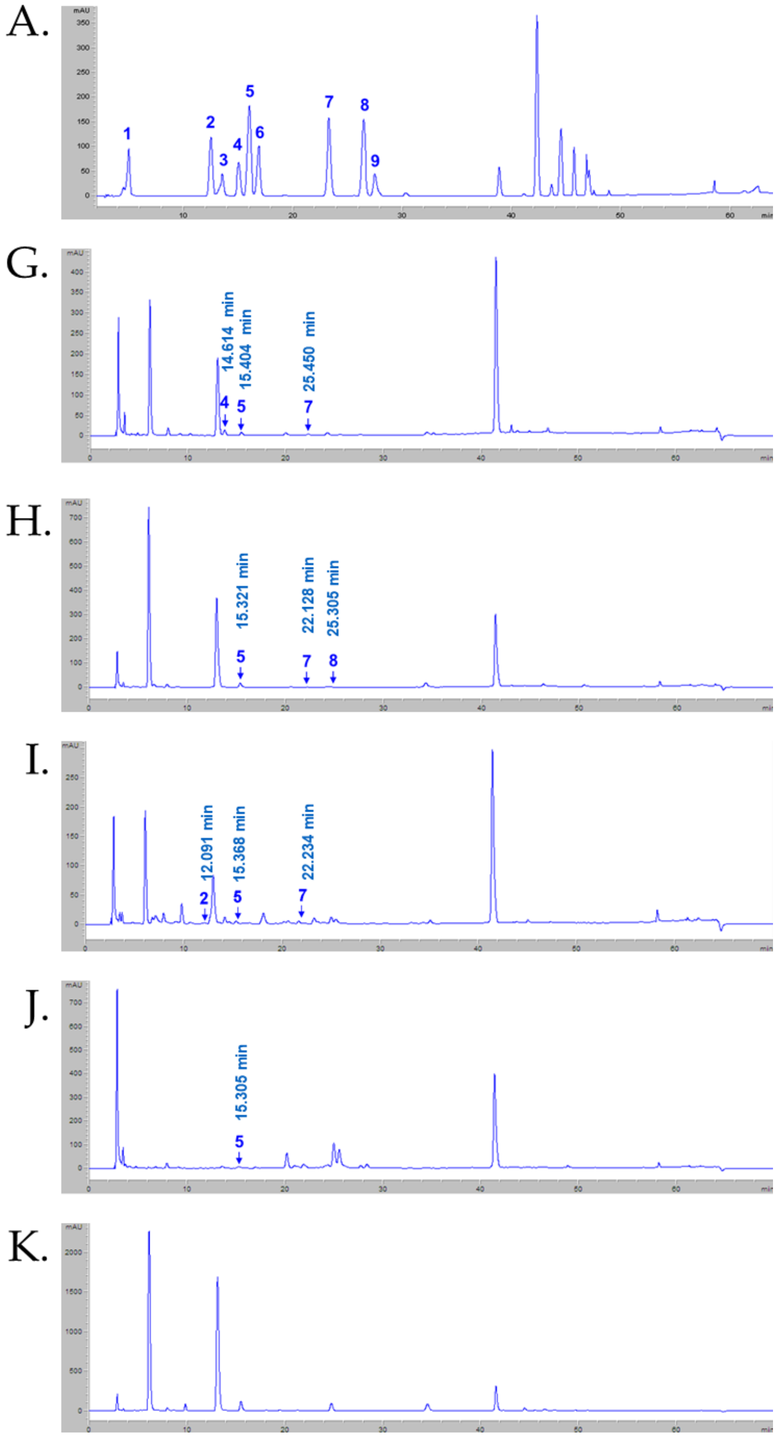

1 = gallic acid (4.8 min)
2 = 4-hydroxybenzoic acid (12.1 min)
3 = chlorogenic acid (13.3 min)
4 = vanillic acid (14.6 min)
5 = caffeic acid (15.3 min)
6 = syringic acid (16.5 min)
7 = *p*-coumaric acid (22.4 min)
8 = ferulic acid (22.5 min)
9 = sinapic acid (26.5)

Supplementary Figure S2:

High-performance liquid chromatograms showing retention time (*Rt*) of (A) authentic standards including chlorogenic acid (13.3 min), caffeic acid (15.3 min), *p*-coumaric acid (22.2 min), ferulic acid (25.4 min), sinapic acid (26.5 min), myricetin (37.9 min), quercetin (43.1 min), luteolin (43.7 min), hesperitin (45.2 min), kaempferol (46.2 min), apigenin (46.5 min) and isorhamnetin (47.1 min), (B.) *A. cepa*, (C.) *A. fistulosum*, (D.) *A. sativum*, (E.) *Ci. hystrix*, (F.) *Co. sativum*, (G.) *Cy. citratus*, (H.) *E. foetidum*, (I.) *P. tetragonolobus*, (J.) *Se. pennata*, and (K.) *So. melongena* detected at 338 nm.

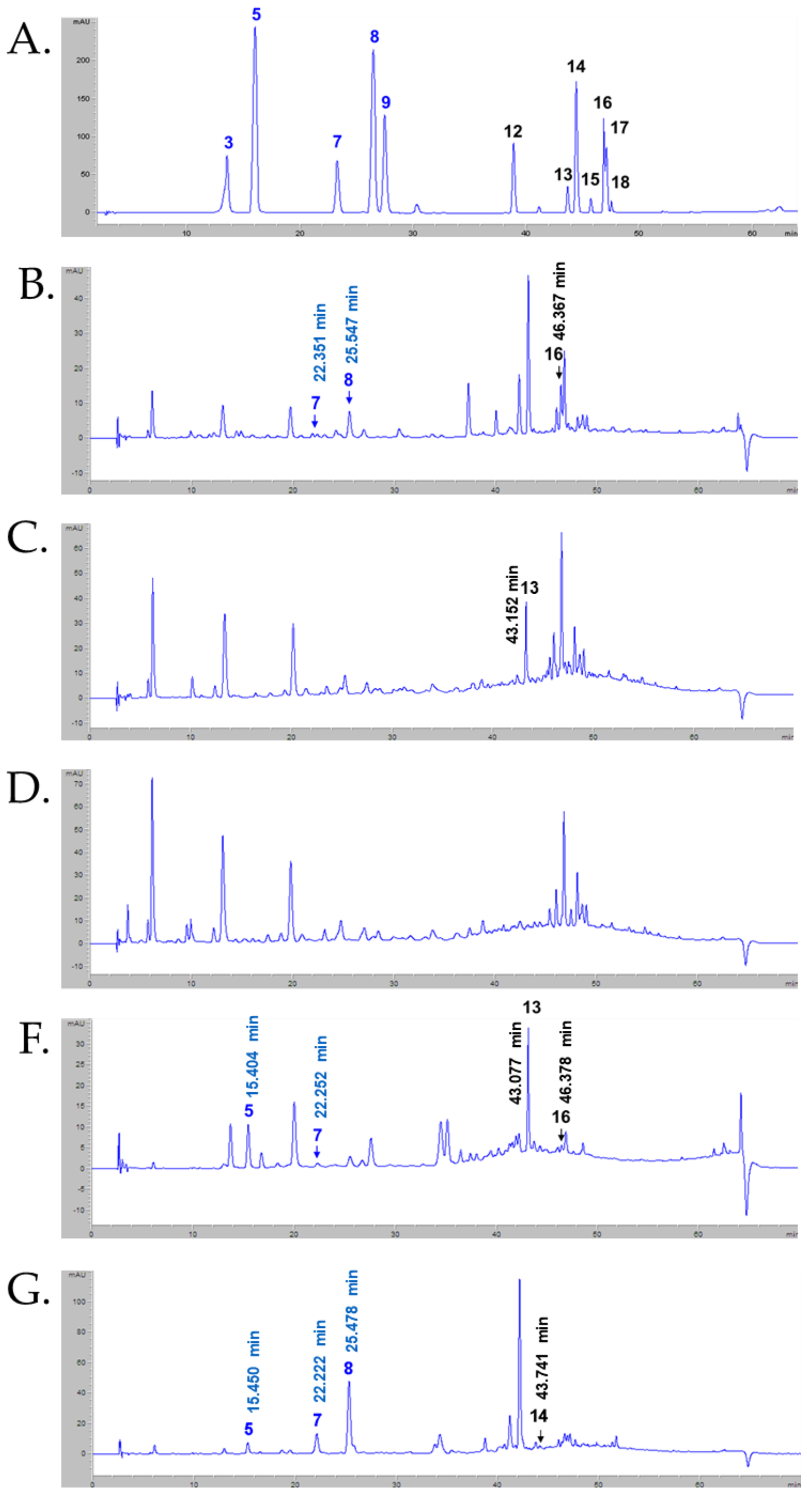

3 = chlorogenic acid (13.3 min)
5 = caffeic acid (15.3 min)
7 = *p*-coumaric acid (22.2 min)
8 = ferulic acid (25.4 min)
9 = sinapic acid (26.5 min)
12 = myricetin (37.9 min)
13 = quercetin (43.1 min)
14 = luteolin (43.7 min)
15 = hesperitin (45.2 min)
16 = kaempferol (46.2 min)
17 = apigenin (46.5 min)
18 = isorhamnetin (47.1 min)

Supplementary Figure S2 (Cont.):

High-performance liquid chromatograms showing retention time (*Rt*) of (A) authentic standards including chlorogenic acid (13.3 min), caffeic acid (15.3 min), *p*-coumaric acid (22.2 min), ferulic acid (25.4 min), sinapic acid (26.5 min), myricetin (37.9 min), quercetin (43.1 min), luteolin (43.7 min), hesperitin (45.2 min), kaempferol (46.2 min), apigenin (46.5 min) and isorhamnetin (47.1 min), (B.) *A. cepa*, (C.) *A. fistulosum*, (D.) *A. sativum*, (E.) *Ci. hystrix*, (F.) *Co. sativum*, (G.) *Cy. citratus*, (H.) *E. foetidum*, (I.) *P. tetragonolobus*, (J.) *Se. pennata*, and (K.) *So. melongena* detected at 338 nm.

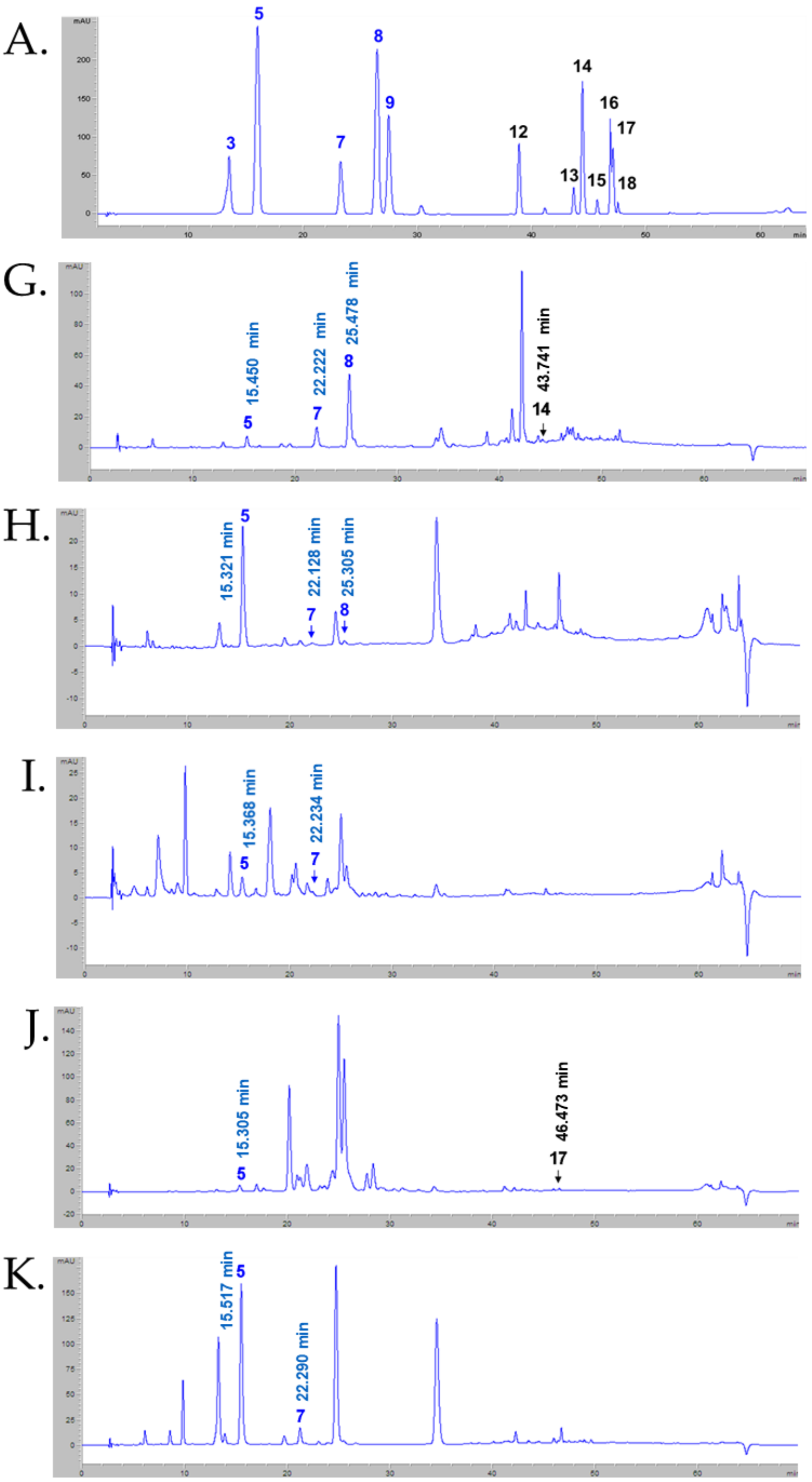

3 = chlorogenic acid (13.3 min)
5 = caffeic acid (15.3 min)
7 = *p*-coumaric acid (22.2 min)
8 = ferulic acid (25.4 min)
9 = sinapic acid (26.5 min)
12 = myricetin (37.9 min)
13 = quercetin (43.1 min)
14 = luteolin (43.7 min)
15 = hesperitin (45.2 min)
16 = kaempferol (46.2 min)
17 = apigenin (46.5 min)
18 = isorhamnetin (47.1 min)

Supplementary Figure S3:

High-performance liquid chromatograms showing retention time (*Rt*) of (A) authentic standards including delphinidin (29.0 min) and cyanidin (35.3 min), (B.) *A. cepa*, (C.) *A. fistulosum*, (D.) *A. sativum*, (E.) *Ci. hystrix*, (F.) *Co. sativum*, (G.) *Cy. citratus*, (H.) *E. foetidum*, (I.) *P. tetragonolobus*, (J.) *Se. pennata*, and (K.) *So. melongena* detected at 524 nm.

A.

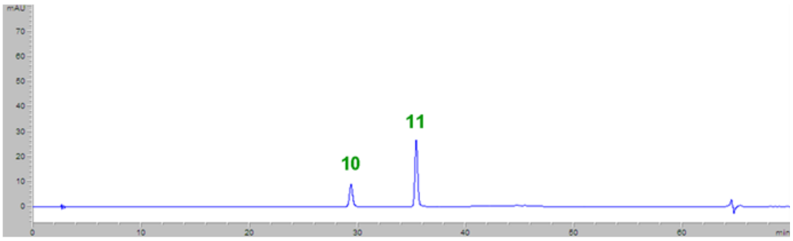

10 = delphinidin (29.0 min)

11 = cyanidin (35.3 min)

B.

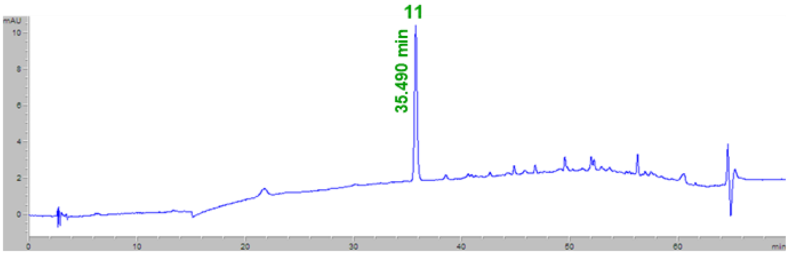

C.

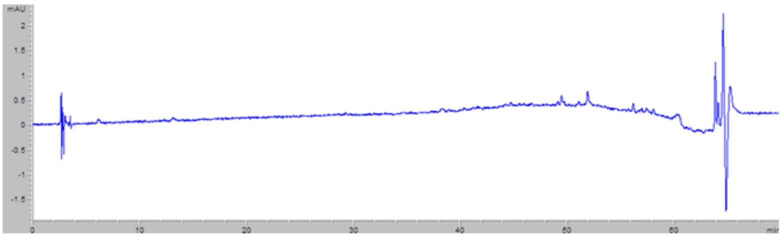

D.

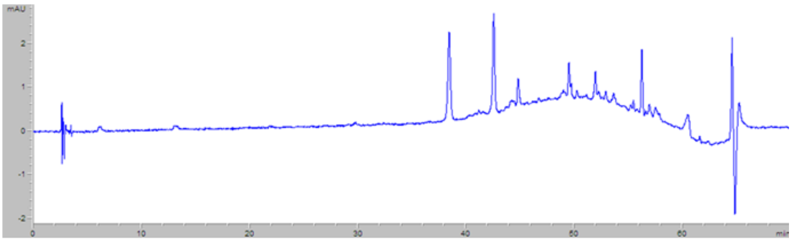

E.

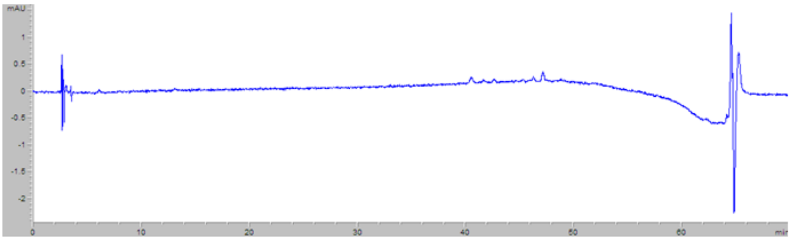

F.

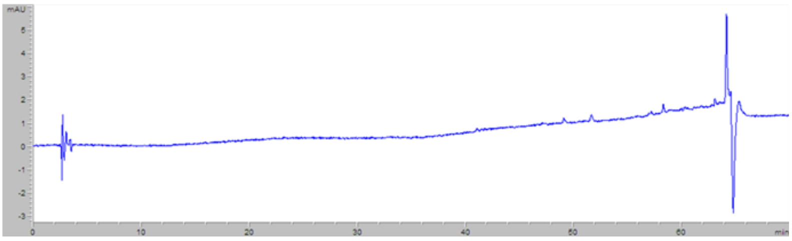

Supplementary Figure S3 (Cont.):

High-performance liquid chromatograms showing retention time (*Rt*) of (A) authentic standards including delphinidin (29.0 min) and cyanidin (35.3 min), (B.) *A. cepa*, (C.) *A. fistulosum*, (D.) *A. sativum*, (E.) *Ci. hystrix*, (F.) *Co. sativum*, (G.) *Cy. citratus*, (H.) *E. foetidum*, (I.) *P. tetragonolobus*, (J.) *Se. pennata*, and (K.) *So. melongena* detected at 524 nm.

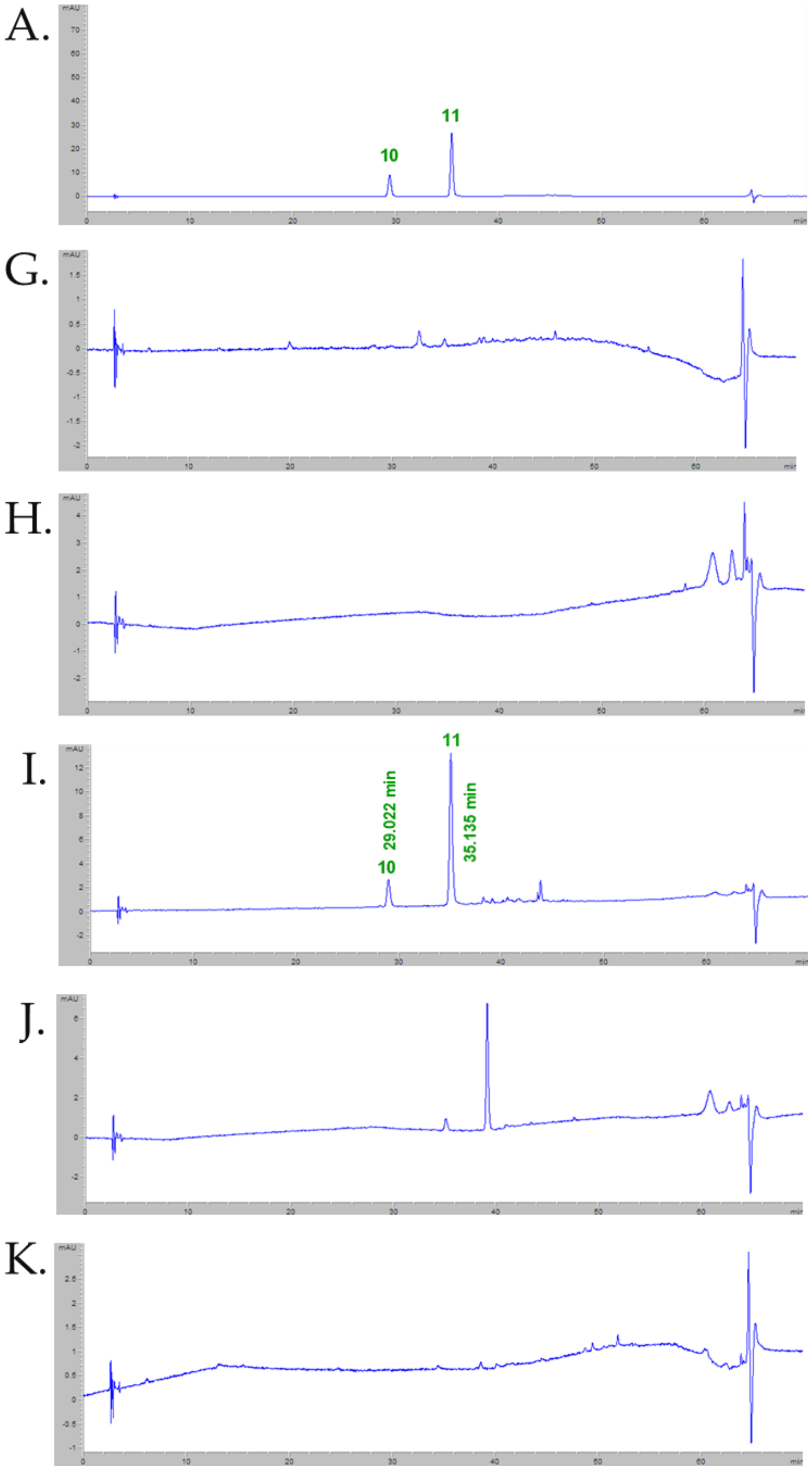

10 = delphinidin (29.0 min)

11 = cyanidin (35.3 min)
